# Microenvironment Tailoring for Electrocatalytic CO_2_ Reduction: Effects of Interfacial Structure on Controlling Activity and Selectivity

**DOI:** 10.1021/jacs.4c13494

**Published:** 2025-03-12

**Authors:** Yaqi Cheng, Qixun Li, Muhammad Iskandar B. Salaman, Chaolong Wei, Qilun Wang, Xuehu Ma, Bin Liu, Andrew Barnabas Wong

**Affiliations:** † Department of Materials Science and Engineering, National University of Singapore, Singapore 117575, Singapore; ‡ Institute of Chemical Engineering, Dalian University of Technology, Dalian 116024, China; § Department of Materials Science and Engineering, City University of Hong Kong, Kowloon, Hong Kong SAR 999077, China; ∥ Department of Chemical and Biomolecular Engineering, National University of Singapore, Singapore 117585, Singapore

## Abstract

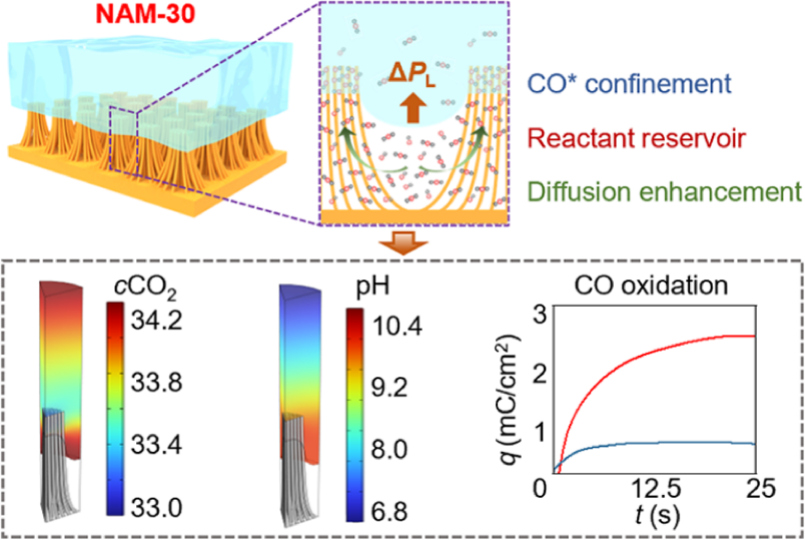

The performance of the electrocatalytic CO_2_ reduction reaction (CO_2_RR) is highly dependent on the microenvironment around the cathode. Despite efforts to optimize the microenvironment by modifying nanostructured catalysts or microporous gas diffusion electrodes, their inherent disorder presents a significant challenge to understanding how interfacial structure arrangement within the electrode governs the microenvironment for CO_2_RR. This knowledge gap limits fundamental understanding of CO_2_RR while also hindering efforts to enhance CO_2_RR selectivity and activity. In this work, we investigate this knowledge gap using a tunable system featuring superhydrophobic hierarchical Cu nanowire arrays with microgrooves (NAMs). Adjusting the NAM structure tunes multiple synergistic effects in the microenvironment, which include stabilization of the microwetting state, confinement of CO*, improvement to local CO_2_ concentration, and modulation of the local pH. Notably, using mass transport modeling, we quantify the role of the gas–liquid–solid interface in boosting local CO_2_ concentrations within several microns of the interface itself. Leveraging these effects, we elucidate how CO* and H* competitively occupy active sites, influencing reaction pathways toward multicarbon products based on tuning the microenvironment. Consequently, we provide new insights into why the optimized configuration significantly increased CO_2_RR activity by 690% (as normalized by electrochemical active surface area), C_2+_ product selectivity by 72%, and Faradaic efficiency by 36%, compared to CO_2_RR with hydrophobic Cu foil. Based on these insights, our findings unlock new opportunities to engineer the CO_2_RR microenvironment through the rational organization of hierarchical interface materials in gas diffusion electrodes toward improved CO_2_RR selectivity and activity.

## Introduction

With the reliance on fossil fuel combustion-based energy systems, carbon dioxide (CO_2_) accumulation in the atmosphere has contributed to a greenhouse effect, driving global climate change, which has drawn increasing attention for the past several decades.^1^ To recycle CO_2_ and mitigate its emissions, the electrochemical carbon dioxide reduction reaction (CO_2_RR) provides an effective strategy for a carbon-neutral future and climate change elimination.^2,3^ Moreover, as an electrocatalytic process, CO_2_RR pairs well with renewable electricity sources, such as solar, tidal, or wind, enabling the sustainable production of value-added fuels and chemicals.^4,5^ In particular, Cu-based catalysts for CO_2_RR have received considerable attention because Cu is uniquely active for carbon–carbon (C–C) coupling to generate multicarbon products, including hydrocarbons and oxygenated products.^6,7^ Consequently, tremendous efforts have focused on designing Cu-based catalysts with optimized adsorbate energies of key intermediates by manipulating their surface morphology,^8−13^ size,^14−18^ grain boundaries,^19−22^ and chemical composition,^23−26^ thereby promoting their intrinsic activity and selectivity in terms of Faradaic efficiency (FE) for CO_2_RR. Beyond the intrinsic properties of catalysts, the local microenvironment can also play a crucial role in determining the activity and product selectivity of CO_2_RR. For example, high local CO_2_ concentration at the electrode surface not only facilitates large production rate, but also endows higher selectivity of C_2+_ products by concentrating the key intermediates.^27,28^ Additionally, morphology-induced higher local pH near the electrode on the one hand inhibit hydrogen evolution reaction, on the other hand can direct the reaction toward the CO pathway for C_2+_ products due to the fewer protons involved.^29−33^ While the microenvironment is critical for determining CO_2_RR performance, systematic approaches to optimize the local microenvironment at the catalyst-electrolyte interface have been elusive. Especially, the connection between the microenvironment and interfacial structure within an electrode remains an area that is relatively poorly understood. This critical knowledge gap limits the ability to rationally adjust the microenvironment to optimize CO_2_RR selectivity and activity by adjusting electrode structure.

Recent studies have demonstrated that incorporating catalyst–electrolyte–gas triple-phase interfaces can concentrate CO_2_ reactants and accommodate a local alkaline environment to enhance the CO_2_RR activity and tune product selectivity.^34−37^ Thus, the implemented strategies have focused on constructing hydrophobic nanostructured surfaces with water-resistant and CO_2_-trapping functionalities,^38−42^ and on employing porous gas diffusion electrodes (GDEs) that have Janus contacts, interfacing with the electrolyte on one side and the flowing CO_2_ gas on the other.^43−45^ Nevertheless, so far, these nanofabricated Cu electrodes and microporous GDEs possess high degrees of disorder as well as high variability in shape and size,^40,41^ making it challenging to quantify how the catalyst structural arrangement regulates the mass transport of reactant species, the CO_2_RR microenvironment, and consequently CO_2_RR performance.^46^ Therefore, model systems are urgently needed. Such model systems should be amenable to 3D transport modeling with tunable structures that can precisely probe the relationship between the structure, interfaces, transport, microenvironment, and performance to gain additional insights into the design principles of catalyst arrangement and GDEs toward the ultimate goal of understanding how CO_2_RR performance can improve in terms of both selectivity and activity.

In this work, we unveil the roles of synergistic phenomena within the CO_2_RR microenvironment using our tunable superhydrophobic nanowire arrays with microgrooves (NAMs) cathodes as model platforms. To characterize the microenvironment in these NAMs model systems, we employ a suite of approaches that include laser scanning confocal microscopy, three-dimensional mass transfer modeling, and electrochemical characterization techniques. The microgrooves together with densely packed nanowires at the top of the bundle of the NAM-30 (NAMs with a microgroove opening angle of 30°) structure exert upward Laplace pressure to stabilize the wetting state of the gas–liquid–solid interface for favorable CO_2_ transport. Based on this, we show increased availability of CO_2_ at two-phase liquid–solid interfaces within several microns of the 3-phase gas–liquid–solid interface, allowing Cu sites at both interfaces to have more access to CO_2_. Moreover, the superior CO* confinement at the top of nanowire bundles also leads to more facile C–C coupling to form C_2_ products. In addition to this, we find that adjusting the structure of the NAM-based cathode can tune the local pH, which can allow H* to participate in hydrogenation steps within CO_2_RR rather than HER in the presence of improved availability of CO_2_ and CO. Taken together, we show that tuning the structure of the cathode interfaces can tailor the CO_2_RR microenvironment to achieve improved CO_2_RR performance. These performance improvements include a 690% increase in the electrochemical active surface area (ECSA)-normalized activity, a 72% increase in the C_2+_ product selectivity, and a 36% increase in the Faradaic efficiency for CO_2_RR as compared to the CO_2_RR performance of hydrophobic Cu foil. These insights reveal essential new design concepts for improving CO_2_RR selectivity and activity by tuning the CO_2_RR microenvironment, which informs efforts to improve CO_2_RR performance and scalability.

## Results

### Design and Characterization of NAMs

**Figure 1:**
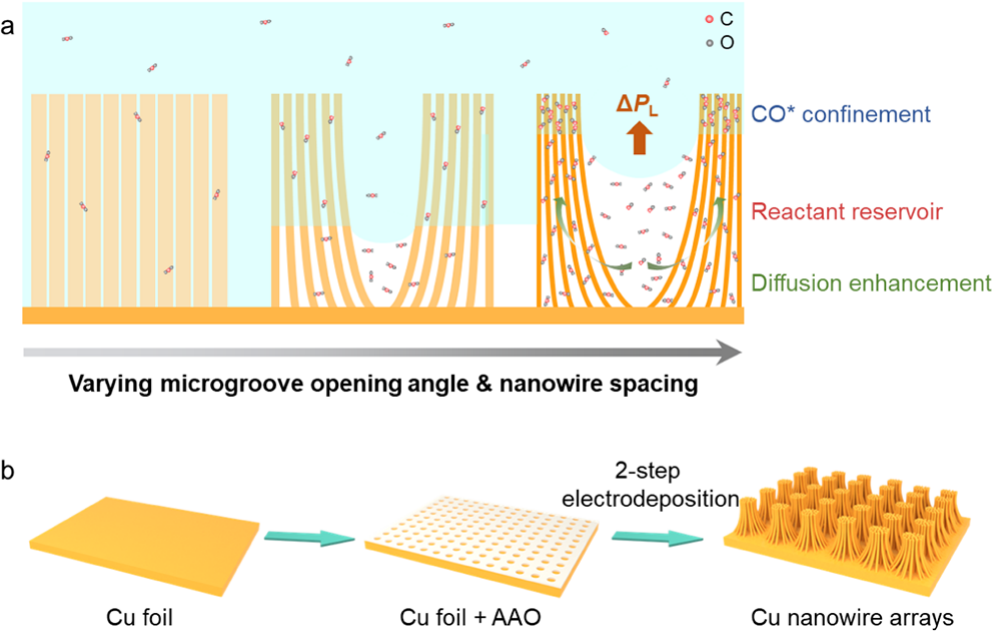
Schematic illustration of the tunable hierarchical structure design and corresponding preparation strategy for enhancing CO_2_ mass transfer and optimizing the local microenvironment. (a) Liquid–solid–gas three-phase schematic highlighting the tunable hierarchically structures, the wetting state and the adaptively changed mass transport. (b) Schematic illustration of the realization method of hierarchically structured NAMs.

Figure 1a shows a schematic drawing of our designed tunable hierarchical model structure, which consists of microgrooves and gradient nanostructures. Solely by adjusting the diameter of the nanowires, both the spacing between nanowires and the opening angle of the microgrooves can be tuned accordingly because of the soft nature of the Cu nanowires that can bend and agglomerate into nanowire bundles. Based on functionality, we divide the structure into three zones: the microgroove, the densely packed nanostructure at the top, and the sparsely arranged nanostructure at the bottom. The microgrooves and nanowires exert upward Laplace pressures up to 76 and 72 kPa respectively (see Figure S1, detailed calculation and more insights into the role of nanowire length are presented in Supporting Information), together to stabilize the electrolyte–gas interface for a stable Cassie–Baxter wetting state,^47^ which not only enhances gaseous reactant diffusion from the bottom gas layer to the top reaction zone but also traps the produced CO in the gas pocket. The sparsely arranged nanostructure at the bottom is designed to reduce the lateral mass transfer resistance of the gaseous reactant. Conversely, the densely packed nanostructures at the top synergistically confine the CO* and promote C–C coupling for multicarbon products.

We fabricated superhydrophobic nanowire arrays with microgrooves (NAMs) through a two-step template-assisted electrodeposition method,^48^ followed by a hydrophobic treatment. The template assisted electrodeposition is depicted in Figure 1b. In the first step, the anodic alumina oxide (AAO) template is attached onto a planar Cu foil by electrodeposition at −0.8 V for 60 min, as shown in Figure S2. During this electrodeposition process, short nanowires are formed in the holes of AAO to fix the AAO template on the Cu surface. In the second step, the nanowires grow longer by electrodeposition for another 60 min. Then, the AAO template is removed by immersion in a 2 M NaOH solution. Finally, treatment with an *n*-octadecanethiol solution confers superhydrophobicity to the samples. In this discussion, “NAMs” refer to samples that have undergone hydrophobic treatment, while “Hy-NAMs” denote samples prior to hydrophobic treatment. Similarly, “Cu foil” refers to hydrophobic copper foil, whereas “Hy-Cu” refers to copper foil before hydrophobic treatment.

**Figure 2:**
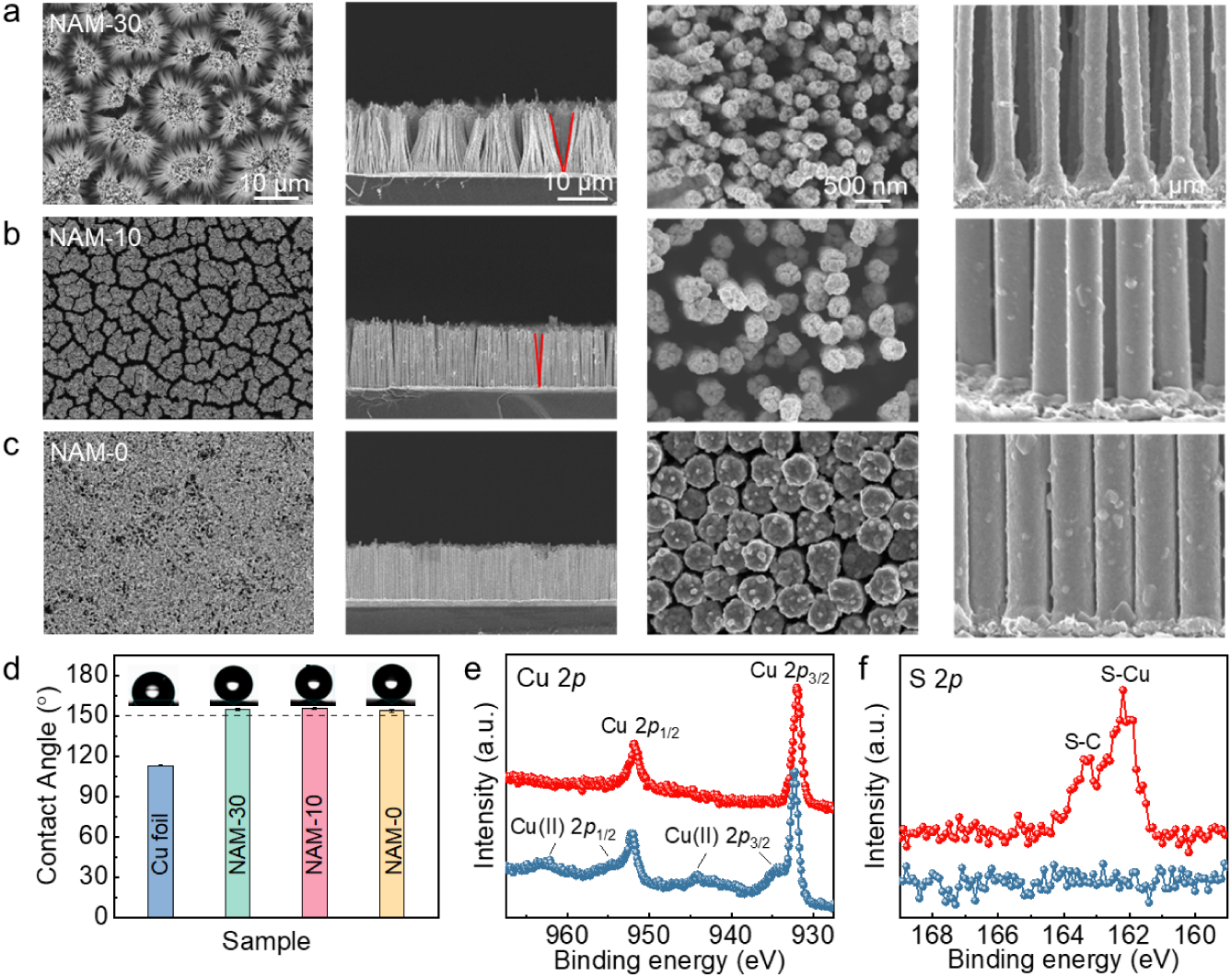
Characterization of NAMs. Scanning electron microscopy (SEM) images of NAMs with opening angles of (a) 30°, (b) 10°, and (c) 0° for the microgrooves. The first and second columns show the nanowire arrays with microgrooves from the top and side views, respectively. The third and fourth columns show the densely packed nanowires at the top and sparsely arranged nanowires at the bottom, respectively. (d)The contact angles for the NAMs and Cu foil. (e) High-resolution XPS Cu 2*p* spectra of NAMs before (blue) and after (red) hydrophobic treatment. (f) High-resolution XPS S 2*p* spectra of NAMs before (blue) and after (red) hydrophobic treatment.

Our study employed three variants of commercial AAO templates to fabricate nanowire arrays with different structural parameters. As depicted in the SEM images of these AAO templates (see Figure S3), the pores of all the AAO templates show a hexagonal pattern with the same pore pitch of approximately 450 nm but varying pore diameters measuring 200, 300, and 400 nm, respectively. The geometries of the as-fabricated NAMs with different structural parameters are shown in Figure 2a–c. The nanowires undergo bending and aggregation due to the surface tension of water upon removal from the NaOH solution. Consequently, nanowire bundles and microgrooves form, leading to a notable gradient structure in that the nanowires at the top are densely packed, while those at the bottom are sparsely arranged, as shown in Figure 2a. As the diameter of the nanowire increases, its rigidity improves, leading to a decrease in both the opening angle of the microgrooves and the spacing between the bottom nanowires. In contrast, the spacings between the nanowires at the top remain comparable, as shown in Figure 2b,c. The NAMs with nanowire diameters of 200, 300, and 400 nm exhibit opening angles of ∼30° (28.5 ± 1.8°), 10° (12.2 ± 0.7°), and 0° (0 ± 0°), respectively, for the microgrooves and are thus termed NAM-30, NAM-10, and NAM-0, respectively. Before hydrophobic treatment, all samples are hydrophilic, having contact angles smaller than 90° (see Figure S4). However, after hydrophobic treatment, the materials exhibit superhydrophobicity, with contact angles exceeding 150°, as shown in Figure 2d. Furthermore, X-ray diffraction (XRD) patterns are explored. As all the samples exhibit identical XRD characteristics, we have chosen NAM-30 as a representative example for the demonstration (see Figure S5). The results show that all the samples have the same three peaks at 43.3°, 50.4°, and 74.1°, corresponding to face-centered cubic Cu(111), Cu(200), and Cu(220), respectively, which demonstrates that the electrodeposition and hydrophobic treatment processes do not alter the crystal structure of Cu. In addition, the hydroxyl underpotential deposition (UPD) is carried out to assess the surface termination of the NAMs (Figure S6). The cyclic voltammetry of NAMs shows the same adsorption peak at −670 mV and its counterpart at −680 mV, indicating that the nanowires within the NAMs possess some population of Cu(100) facets.^49^

The chemical states of the NAMs were further characterized by X-ray photoelectron spectroscopy (XPS), as shown in Figures 2e and S7. All samples exhibit the same Cu(0/I) state, which is evident as two peaks at 933.3 (Cu 2p_3/2_) and 952.2 eV (Cu 2p_1/2_). It should be noted that the minor peaks at 934.5/944.1 eV and 955.0/962.8 eV for the hydrophilic NAMs can be attributed to Cu(II) 2p_3/2_ and Cu(II) 2p_1/2_, respectively. However, after the hydrophobic treatment involving alkanethiolation, Cu(II) oxide is removed from the surface, leaving Cu(I)–S bonds,^50^ which is consistent with the peak of S 2p at 162.2 eV shown in Figure 2f. The energy dispersive spectroscopy (EDS) mapping of NAM-30 is also shown in Figure S8, which is consistent with the XPS results. All of these results imply that the designed NAMs possess homogeneous physical and chemical properties while the varying structural parameters were successfully obtained.

### Electrochemical CO_2_ Conversion

**Figure 3:**
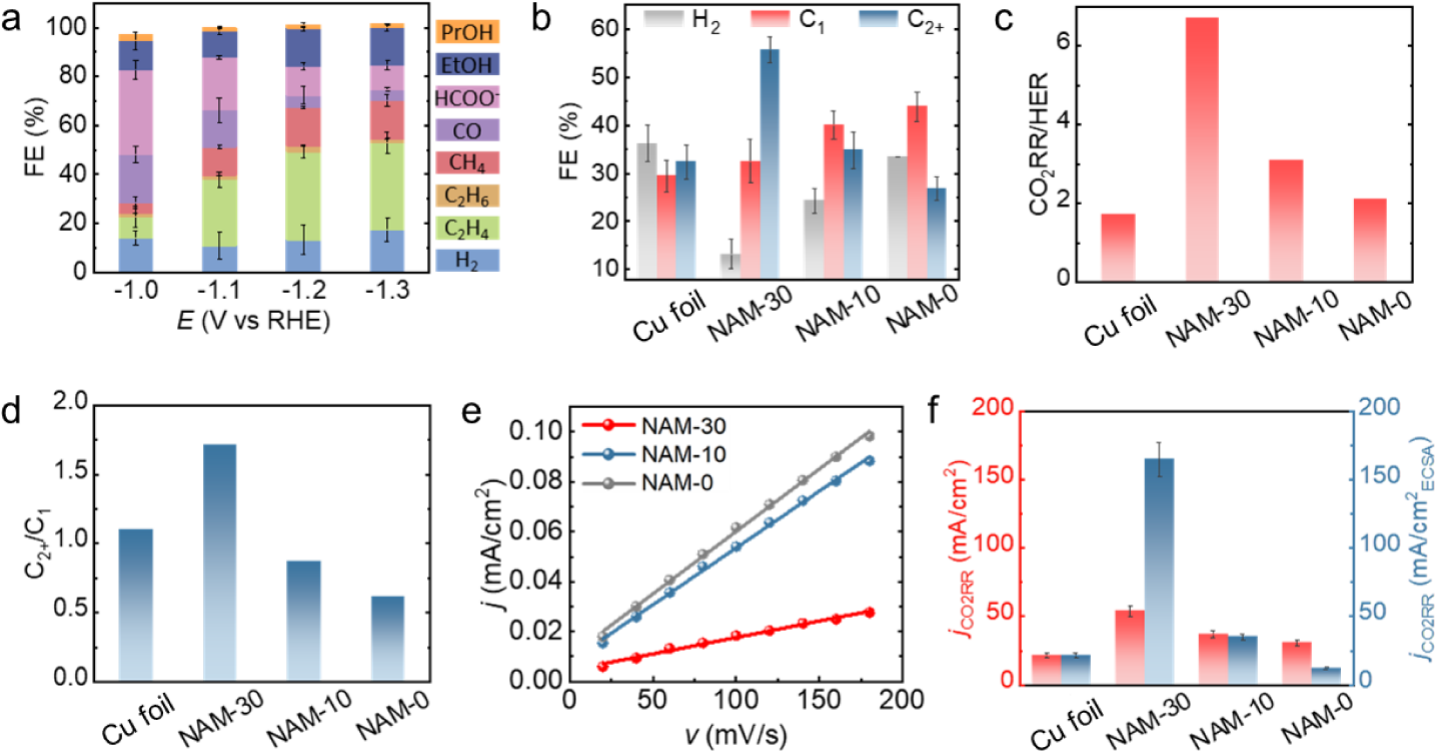
CO_2_RR performance of NAMs in a CO_2_-saturated 0.1 M KHCO_3_ electrolyte. (a) Product selectivity of NAM-30 as a function of applied potential. (b) Product distribution of all samples at −1.2 V vs RHE. (c) Ratio of carbon reduction products to hydrogen at −1.2 V vs RHE. (d) Ratio of C_2+_ products to C_1_ products at −1.2 V vs RHE. (e) Calculation of *C*_dl_ based on the current densities and scan rates of the corresponding cyclic voltammetry (CV) curves. (f) Comparison of the geometric area- and ECSA-normalized partial current density of CO_2_RR for all samples at −1.2 V vs RHE. The ECSA data are collected after 120 min of CO_2_RR, during which the ECSA stabilizes.

To evaluate the influence of the structure on the electrocatalytic performance of NAMs, the electrochemical reduction of CO_2_ was investigated in an H-cell with a three-electrode configuration, where Ag/AgCl was the reference electrode, Pt foil was the counter electrode, and CO_2_-saturated 0.1 M KHCO_3_ was the electrolyte. The FEs presented in Figure 3a reflect the products at applied potentials between −1.0 and −1.3 V vs the reversible hydrogen electrode (RHE) for NAM-30. In this chosen potential range, the NAMs are stable during our CO_2_RR experiments, and we avoid intrinsic wettability changes that would be predicted at more reducing potentials.^51−54^ The selectivity of NAM-10, NAM-0, Cu foil, and Hy-NAM-30 is depicted in Figure S9. The results show a significant suppression of HER and enhanced CO_2_RR when comparing NAM-30 to Hy-NAM-30, which can be attributed to the improved hydrophobicity. With increasingly negative applied potentials, the FEs of CO and HCOO^–^ decrease, while the FEs of CH_4_, C_2_H_4_, and CH_3_CH_2_OH increase. Although all three NAMs achieve their optimal total FE for the CO_2_RR at −1.1 V vs RHE, this value is further improved with the increased prominence of microgrooves, as NAM-30 achieves the highest FE for CO_2_RR at 90.3%. Specifically, as the most valuable products, the FEs of C_2+_ compounds exhibit the highest values at −1.2 V vs RHE for all NAMs. For a detailed comparison, the CO_2_RR selectivity among all NAMs and Cu foil is depicted in Figure 3b. The FEs of the H_2_ and C_1_ products increase when the opening angle of the grooves decreases, whereas the FEs of the C_2+_ products show the opposite trend. Consequently, NAM-30 achieves the lowest FE of H_2_ (13.1%) and the highest FE of C_2+_ products (55.7%), respectively, indicating a 64% improvement in reducing the FE to competing reaction (i.e., hydrogen evolution reaction (HER)) suppression and a 72% improvement in C_2+_ production when compared to the Cu foil. In addition, NAM-30 exhibits enhanced selectivity for C_2+_ products when compared to Cu nanowires with similar facet types and reactor configurations in prior studies (Table S1). In fact, the CO_2_RR-to-HER ratio is dramatically increased from 2.1 to 6.7 by increasing the opening angle of the microgrooves and the bottom nanowire spacing, as shown in Figure 3c. Simultaneously, the C_2+_-to-C_1_ ratio is also effectively tuned from 0.6 to 1.7 by optimizing the structural parameters of the NAMs (Figure 3d). These results suggest that the arrangement of catalyst structures plays a critical role in tuning the selectivity of CO_2_RR; the increased prominence of microgrooves and the bottom nanowire spacing not only inhibits HER but also directs the reaction pathways toward C_2+_ products.

In addition to selectivity, another important metric in CO_2_RR is the current density (current normalized by the geometric electrode area, 1 cm^2^), especially the partial current density, as presented in Figures S10 and S11. Interestingly, the partial current densities of CO_2_RR and HER also strongly depend on the structural geometries of the samples. Compared to that of Cu foil, the partial current density of NAM-30 exhibited a 145% improvement in CO_2_RR and 50% suppression in HER. The superhydrophobic wettability facilitates gas trapping beneath the nanowires, improving gas-phase CO_2_ reactant supply in CO_2_RR and reducing the contact area between the electrode and electrolyte liquid for HER.

To further verify this observation, we measured the electrochemical active surface area (ECSA) of the NAMs. Figure S12 displays the corresponding cyclic voltammetry (CV) curves, while the calculated *C*_dl_ and ECSA based on the current values and the scan rates are listed in Table S2. As shown in Figure 3e, the ECSA decreases with increasing opening angle of the microgrooves, demonstrating a smaller overall contact area between the electrode surface and the electrolyte owing to the replacement of trapped gas within the nanowire arrays. Furthermore, the ECSA-normalized current density of CO_2_RR on NAM-30 significantly increased to 162 mA/cm^2^_ECSA_, demonstrating a 7.9-fold enhancement compared with that of Cu foil (Figure 3f). On the other hand, for NAM-0, even though the geometric area-normalized partial current density of CO_2_RR is greater than that of Cu foil, the lower ECSA-normalized partial current density implies that the better apparent activity originates from its enlarged contact area rather than from the increase in the intrinsic activity.^55^

The electrochemical stability of NAM-30 was evaluated, and the results are presented in Figure S13. The data demonstrate that both the current density and FE remain consistent, indicating the excellent stability of NAM-30 during a 180 min CO_2_RR test. Additionally, the SEM, UPD, XRD, and XPS analyses shown in Figures S14–S17 reveal no significant changes in the overall morphology, the facets, and chemical states for all NAMs throughout the CO_2_RR process. Besides, the evolution of the ECSA over time, as illustrated in Figures S18 and S19, indicates a notable change during the initial 10 min of the reaction but gradually stabilizes and remains relatively constant over a 120 min CO_2_RR period. This variation is attributed to the establishment of equilibrium within the three-phase contact region involving the nanowire arrays, electrolyte, and gas phase, as well as the dynamic adsorption and desorption of active species on the catalyst surface in the initial stage of CO_2_RR. These stable behaviors suggest that NAMs maintain good structural integrity during the CO_2_RR process and preserve the stability of the three-phase interface over time.

### Visualization of the Microscopic Wetting State

**Figure 4:**
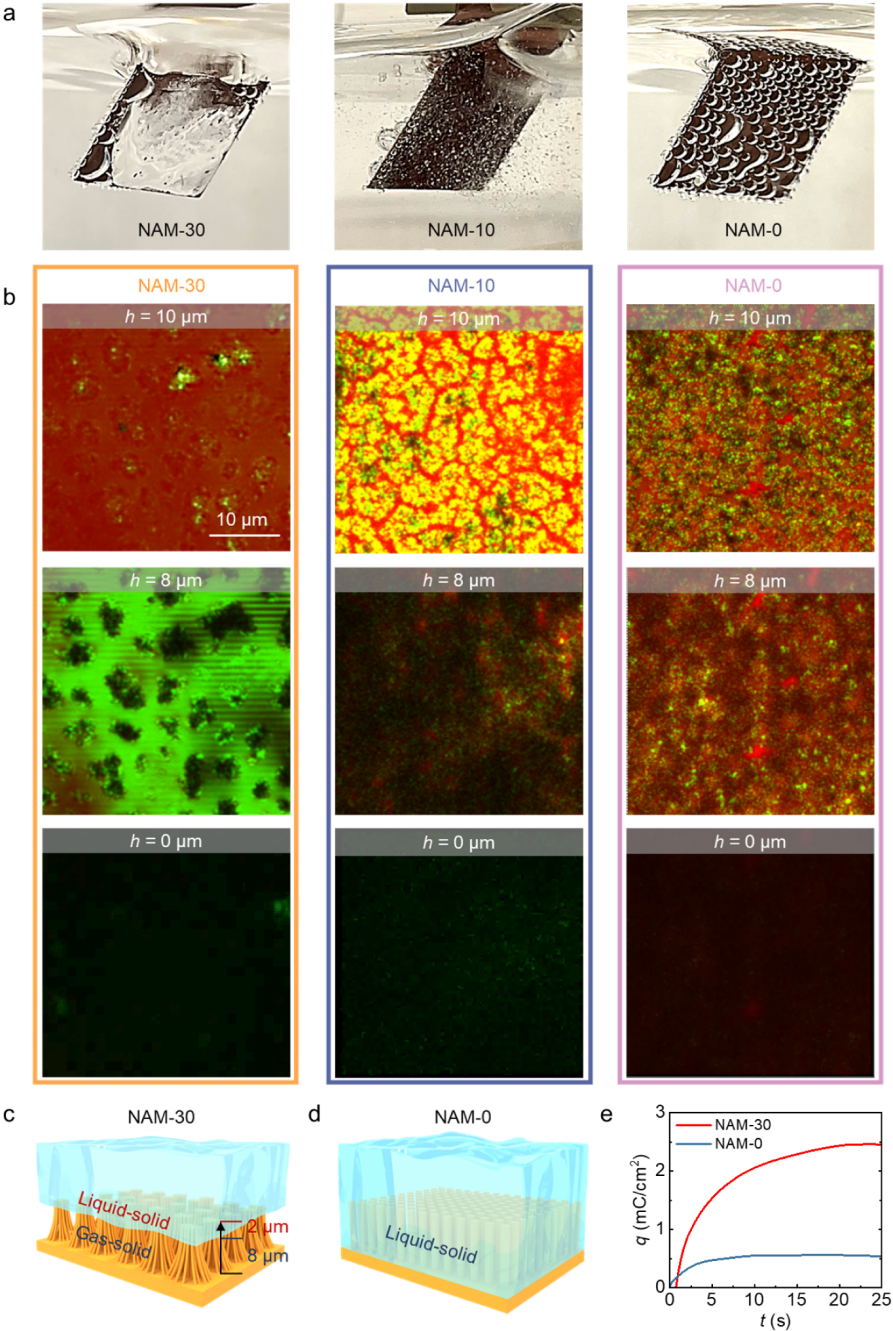
Characterization of gas–liquid–solid interfaces for NAMs during CO_2_RR. (a) Optical observation of the macroscopic wetting states of NAMs in CO_2_RR. (b) Confocal microscopy images of NAM-30, NAM-10, and NAM-0, respectively. The slices are displayed from a top view and selected along the height direction of the nanowires. The red signal represents the emission light of the rhodamine dissolved in the electrolyte, while the green signal is the reflection of the excitation laser light from gas–liquid–solid interfaces as visualized after 2 h into CO_2_RR. (c,d) Schematic illustration of the gas–liquid–solid three-phase interfaces of NAM-30 and NAM-0 in CO_2_RR. (e) Charge of CO oxidation derived from chronoamperometric curves.

To contextualize the correlation between NAM structure and CO_2_RR performance, we have further investigated the wetting state of each NAM to understand the nature of the microenvironment during CO_2_RR conditions. Although all the as-fabricated NAMs are nominally superhydrophobic, they exhibit different macroscopic wetting states during the CO_2_RR process, as shown in Figure 4a. For NAM-30, a gas layer is trapped between the electrode and electrolyte. This demonstrates its stable superhydrophobicity, which sustains a macroscale Cassie–Baxter wetting state during the reaction. In contrast, in the cases of NAM-10 and NAM-0, separated small and large bubbles instead of a continuous gas layer form at the surface, indicating a wetting transition from the Cassie–Baxter state to the Wenzel state.

To further visualize the microscopic wetting state of the NAMs, we observed the shape of the gas–liquid–solid interfaces using laser scanning confocal microscopy after 2 h into the CO_2_RR experiment, as shown in Figure 4b. For NAM-30, when the focus is moving from the top of the nanowire arrays (height from the substrate (*h*) = 10 μm) down to *h* = 8 μm, a strong reflection of the liquid–gas interface located among the nanowire bundles appears, confirming that the liquid–gas interface is continuous and very stable with an approximately 2 μm intrusion depth of the electrolyte into the nanowire arrays. However, the same region of NAM-10 is partially flooded with a discontinuous liquid–gas interface, and the electrolyte is flooded throughout the nanowire arrays for NAM-0. At the bottom of the nanowire arrays, the primary signal is from the reflection of the Cu base for NAM-30 and NAM-10 due to the absence of electrolyte, which is distinguished from the Wenzel wetting state as evidenced by the complete red color of the dyed electrolyte in NAM-0.

The different statuses of the gas–liquid–solid interfaces in the nanowire arrays of NAM-30 and NAM-0 are schematically illustrated in Figure 4c,d, respectively. NAM-30 is in a stable Cassie–Baxter wetting state, with a gas layer in a thickness of 8 μm trapped between the electrolyte and nanowire arrays during CO_2_RR. In contrast, NAM-0 exhibited a Wenzel state, where the electrolyte completely wetted the nanowire arrays. As a transition state between the Cassie–Baxter and Wenzel states, NAM-10 is characterized by a nonuniform partial wetting state. The drastic difference between wetting states in each NAM sample has important implications for the CO_2_RR microenvironment.

### CO Confinement Effect

Water is a critical source of the H* intermediate for both HER and CO_2_RR.^56^ Very recently, during the preparation of the present work, it has been shown that the availability of H* can be effectively tailored by controlling the microwetting state.^57^ In addition, the availability of another intermediate (i.e., CO*) also plays a dominant role in determining the C–C coupling and hence the C_2+_ product selectivity, which is then quantified by in situ CO stripping (i.e., the CO source is produced by CO_2_RR). The CO stripping curves presented in Figure S20 reveal that CO oxidation occurs at ∼0.2 V vs RHE for both NAM-30 and NAM-0.^58^ However, NAM-30 exhibited a significantly greater peak intensity for CO oxidation than did NAM-0. This enhanced performance is even more pronounced when normalized by the ECSA. As shown in Figure S21, NAM-30 shows an impressive 18-fold increase in CO density compared to NAM-0. To gain a more comprehensive understanding, CO stripping chronoamperometry (Figure S22) was then employed to reveal the CO availability via both spatial and temporal metrics. The CO stripping chronoamperometry results before and after the double layer charge correction are shown in Figures S23 and S24, respectively. As expected, the depletion time of CO on NAM-30 (24 s) is 2.2 times that on NAM-0 (11 s), owing to the enhanced CO supply from the confined CO. In addition, throughout the entire process, the CO oxidation current density of NAM-30 consistently surpassed that of NAM-0. Taken together, these findings suggest that the NAM-30 structure promotes CO availability. Furthermore, Figure 4e shows a comparison of the charge transferred during CO oxidation between NAM-30 and NAM-0. NAM-30 exhibited a CO oxidation charge of 2.5 mC/cm^2^, which was 4-fold greater than that of NAM-0 (0.5 mC/cm^2^). This substantial increase in CO availability further provides insights into the cause for the marked increase in C_2+_ production observed in NAM-30, as CO availability is well-known to promote C_2+_ product formation.^28,59,60^

### Mass Transport and the Microenvironment in NAMs

**Figure 5:**
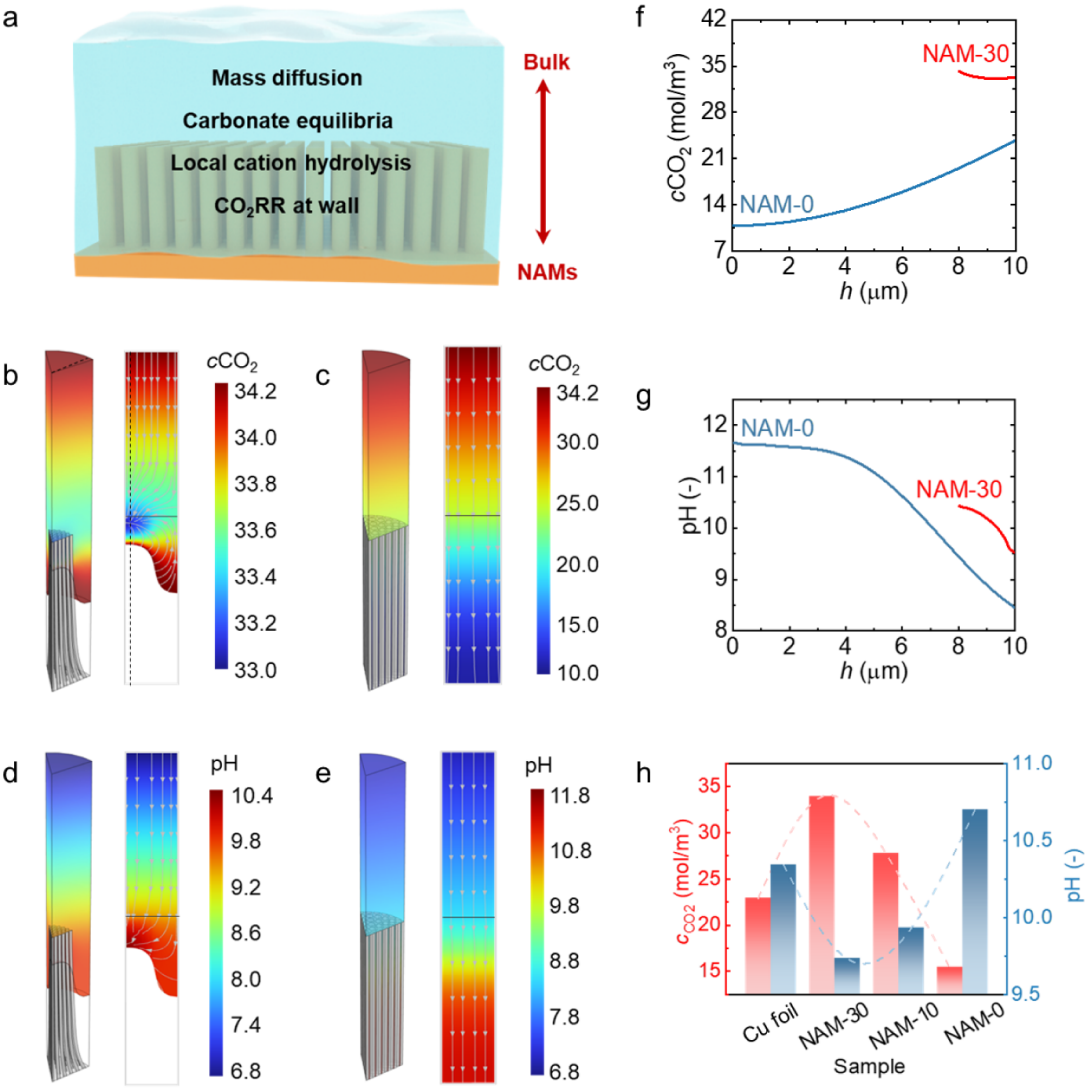
3D numerical simulation of the mass transfer process on NAMs in CO_2_RR. (a) Physical model schematic of the mass transfer processes in CO_2_RR. (b) Contour maps of the CO_2_ concentration profile on NAM-30 in 3D and 2D views of the left and right columns, respectively. The 2D plot is the cross-sectional view labeled by a dashed line in the 3D map. The streamlines indicate the direction of CO_2_ transfer. (c) Contour maps of the CO_2_ concentration profile of NAM-0. (d) Contour maps of the pH of NAM-30. The streamlines indicate the direction of H^+^ transfer. (e) Contour maps of pH on NAM-0. (f) Local concentration distribution of CO_2_ along the height direction of the nanowires. (g) Local pH distribution along the height direction of the nanowires. (h) Comparison of the spatially averaged concentrations of CO_2_ and pH among the NAMs. All the results were acquired at −1.2 V vs RHE.

As mentioned earlier, mass transfer and the microenvironment (e.g., local CO_2_ concentration and pH) around catalysts can significantly affect product distribution and activity.^61−63^ Based on our direct observation of the composite interfaces, we further numerically model in situ three-dimensional (3D) mass transfer processes on NAMs to illustrate the role of the microenvironment in the reaction pathways of CO_2_RR. Herein, we utilized the finite-element method on the COMSOL Multiphysics platform to perform a 3D simulation study. A schematic diagram of the physical model is displayed in Figure 5a. This process involves the mass transport processes, carbonate species equilibria of OH^–^, CO_2_, HCO_3_^–^, and CO_3_^2–^, the local hydrolysis of K^+^ in the electrolyte phase, and mass consumption and generation during CO_2_RR.^64,65^ To perform this analysis, we consider the following five assumptions and boundary conditions (Figure S25): (1) the mass transfer processes are in a steady state, and diffusion occurs near the surface with negligible convection. (2) The CO_2_RR occurs at the liquid–solid interface, so the CO_2_RR-induced mass consumption (CO_2_) and generation (OH^–^) can be represented by a reaction boundary at this interface; thus, electron transfer and mass conservation are correlated with the measured current density and FEs. A zero-flux condition is imposed on nonreactive species at the liquid–solid interface. (3) The top boundary is treated with a constant species concentration as that of the bulk conditions. (4) The boundary of the gas–liquid interface for NAM-30 is regarded with a constant concentration of CO_2_, as a stable CO_2_ gas layer is underneath its nanowires. The detailed assumptions, boundary conditions, settings, and model parameters are provided in “Computational Modeling” in the Experiemental Methods section and Tables S2–S5.

The computational results of the spatial distributions of the CO_2_ concentration and pH for NAMs with different interfacial structures are shown in Figures 5b–e and S26–S28. The CO_2_ concentration and pH data extracted from the contour maps are further intuitively compared in Figure 5f,g. Enabled by the Cassie–Baxter wetting state of NAM-30, bidirectional CO_2_ transport from both the bottom gas layer and the top bulk to the nanowires is observed, as shown by the streamlines in Figure 5b. Compared with the nanowire bundles, the microgrooves show significantly reduced CO_2_ transport resistance, enabling a small CO_2_ concentration gradient and a large CO_2_ concentration (>33.5 mol/m^3^) in the grooves. Consequently, the small diffusion length from the microgroove to the central nanowire bundle further facilitates rapid lateral diffusion and a high local CO_2_ concentration throughout the whole nanowire reactive interface, with a value of at least 33.0 mol/m^3^. Conversely, the Wenzel wetting state of NAM-0 coupled with the narrow spacing between the nanowires results in a near one-directional transport of CO_2_ (Figure 5c). The CO_2_ concentration in NAM-0 was lower than that in NAM-30, with a larger concentration gradient. The highest and lowest concentration values for NAM-0 were 24.3 and 10.0 mol/m^3^, respectively. Overall, this analysis reveals the favorable role of the gas-phase interface within the cathode in increasing the local CO_2_ concentration in the proximity of the gas–liquid–solid interface, in addition to only the specific location of the interface itself. This result is of relevance to the ongoing debate regarding the role of three-phase (gas–liquid–solid) versus two-phase (liquid–solid) interfaces in CO_2_RR.^66−68^

Additionally, in terms of local pH, NAM-30 also has a moderate pH with a slight variation between 9.6 and 10.4 (Figure 5d), which can be ascribed to the short diffusion distance from the gas–liquid interface to the top of the nanowires. However, for NAM-0, both the near one-dimensional and longer diffusion distances result in a large pH gradient ranging from 8.5 (top) to 11.7 (bottom) along the nanowire height (Figure 5e). Therefore, the simulation results highlight the effect of the interfacial structure on both the spatial distribution of the CO_2_ concentration and the pH, and both of these gradients are important aspects of the microenvironment that can determine selectivity and activity for CO_2_RR.

Based on these deep quantitative insights into the local microenvironment, we can determine the underlying causes for the selectivity trends observed in our CO_2_RR performance data. Here, the spatially averaged CO_2_ concentration and pH in the 0 < *h* < 10 μm zone for NAMs were selected as representative descriptors (Figure 5h) to deduce the correlation between the microenvironment and reaction pathways for different products. It has been widely accepted that the concentration of CO_2_ at the reactive interface influences CO_2_ adsorption and that concurrent proton–electron coupled transfers lead to the formation of the key CO* intermediate,^57,69^ which subsequently desorbs from the surface to form CO or further undergoes C–C coupling and protonation steps to form C_2+_ products. On the other hand, the local pH determines the rate of water dissociation and the formation of H*, whose competitive occupation with CO_2_RR intermediates on active sites and influences the electrochemical pathway that occurs, which is responsible for the total FE and variations in CO_2_RR selectivity. Therefore, it is reasonable that the variations in CO* and H* availability caused by the local concentration of CO_2_ and pH via wetting state change is one of the critical reasons for the observed selectivity. First, the high local concentration of CO_2_ and CO* in NAM-30 enables a high occupation ratio of Cu active sites by CO_2_ product intermediates like CO*, which inhibits competing H* adsorption and hydrogen production, in good agreement with the lowest FE of HER occurring on NAM-30. Subsequently, the CO* prefers to undergo protonation as part of the hydrocarbons production rather than being directly desorbed from Cu-based catalysts due to a more neutral environment induced by NAM-30, because a moderate pH is beneficial for electron–proton transfer to access the CHOH* intermediate, which is consistent with recent reports on CO reduction and the mechanistic prediction of pH effects.^70,71^ The results also imply that under these conditions when the CO_2_ concentration is high, H* preferentially participates in protonation in CO_2_RR rather than HER. This is also consistent with recent results showing a tendency for CO_2_RR exhibit higher selectivity versus water reduction as the pH value becomes more neutral.^72^ More importantly, the easy access to high CO* coverage derived from the high local CO_2_ concentration is a crucial factor for C–C coupling, contributing to the improved FE for C_2+_ products on NAM-30.^59^ In contrast, NAM-0, which has the lowest CO_2_ concentration and the highest local pH, has the highest selectivity for HCOO^–^. Here, NAM-0 has the lowest FE of C_2+_ products because of insufficient CO* intermediate density and limited proton availability. Furthermore, the CO_2_ concentration and pH of the Cu foil and NAM-10, together with their CO_2_RR selectivity, are also consistent with these trends. These results imply that the competition for both the spatial occupation of active sites and the subsequent reaction pathway followed by H* are highly dependent on the local CO_2_ concentration and pH. This finding underscores the importance of these factors and their synergistic interactions in designing the arrangement and interface of catalysts, support materials, and electrodes for efficient electrocatalytic CO_2_ conversion. These insights can inform efforts to boost the performance of CO_2_RR in applied systems in terms of selectivity and activity.

## Conclusions

In summary, this work clearly demonstrates the tunability of CO_2_RR activity and selectivity by tailoring the reaction microenvironment near the catalyst surface by adjusting the interfacial structure of the catalyst. To achieve this, we developed and modeled a quasiperiodic system with hierarchically structured nanowire arrays that are encircled by microgrooves, and this hierarchical structure can overcome previous challenges posed by the barriers limiting study of disordered electrodes for CO_2_RR. Within this structure, the microgrooves enhance CO_2_ transport to reaction sites, and the nanowire bundles, which possess an internanowire spacing gradient from bottom to top, simultaneously assist the mass transport of reactants and confine CO* intermediates to promote C–C coupling for multicarbon products. As a result, NAM-30 achieved significant enhancements in the ECSA-normalized activity (690%), faradaic efficiency (36%), and C_2+_ product selectivity (72%) for CO_2_RR, when compared to those of Cu foil. Using laser scanning confocal microscopy, we visually demonstrated how this well-controlled hierarchical morphology helps maintain the Cassie–Baxter wetting state and stabilizes the supply of gaseous reactants (such as CO_2_) to the gas–liquid–solid interfaces for NAM-30. The closely packed nanowires in NAM-30, which facilitate the high availability of CO due to the confinement effect, contribute to improved C_2+_ product selectivity, as confirmed by CO stripping experiments. Leveraging the periodicity of the interfacial geometries in our model system, we successfully developed 3D mass transfer models of the NAMs to quantify the relationship between the reaction microenvironment and performance. Notably, our analysis of the mass transfer models reveals the beneficial effect of the gas–liquid–solid interface on boosting CO_2_ concentrations in the local microenvironment within several microns of the interface itself, which can inform the current understanding of the interplay of the gas–liquid–solid and liquid–solid interfaces for CO_2_RR. Combining all of these investigations, we show that several effects influence the competitive spatial occupation of active sites and determine the subsequent reaction pathway of H* and CO*. These observed effects include the high concentration of CO_2_ present in the gas pocket, the high availability of CO facilitated by CO confinement between nanowires, and the moderate local pH in NAM-30. These synergistic effects influence the reaction pathways to enhance selectivity toward C_2+_ products and reduce the competing hydrogen evolution reaction (HER) in NAM-30. This study provides valuable insights into how hierarchical structure arrangements impact interfacial wetting, microenvironment, and (ultimately) CO_2_RR performance. These design principles can be applied to other state-of-the-art catalyst systems and practical gas diffusion layers for CO_2_RR. Furthermore, our combined approach using laser scanning confocal microscopy and 3D finite element simulation is an excellent example of a simple, accurate, and helpful strategy for studying and developing design rules to optimize multiphase catalysis systems. With further advances in methodology, future studies could further validate the mass transfer model and refine measurements of local pH by building on reports employing in situ methods such as in situ surface-enhanced infrared absorption spectroscopy (SEIRAS)^73−75^ and laser scanning confocal microscopy in conjunction with pH-sensitive fluorescent dyes.^76,77^ By integrating electrochemical testing, laser scanning confocal microscopy, 3D mass transfer modeling, and precise local pH measurements, researchers could establish a comprehensive microenvironment investigation platform for electrocatalysis. This platform would provide deeper insights into the dynamic evolution of local microenvironment and unveil the mass transport effect in structure–activity investigations.

## Data Availability

The data that support the findings of this study are available from the corresponding author upon reasonable request.

## Supporting Information Available

Detailed information on the theoretical calculation of Laplace pressure; additional supporting electrochemical performance and characterization data; additional computational results, and details of methods, including sample preparation, sample characterization, electrochemical measurements, product analysis, laser scanning confocal microscopy, and computational modeling (PDF)
