# Supplementary material for: Microenvironment Tailoring for Electrocatalytic CO2 Reduction: Effects of Interfacial Structure on Controlling Activity and Selectivity
Source: J Am Chem Soc. 2025 Mar 12;147(15):12438–48. doi: 10.1021/jacs.4c13494 (PMC12007005; doi:10.1021/jacs.4c13494)
Supplement: Supplementary file 1 — ja4c13494_si_001.pdf [file ja4c13494_si_001.pdf]

Supporting Information for

Microenvironment tailoring for electrocatalytic CO_2 reduction: Effects of interfacial structure on controlling activity and selectivity

Yaqi Cheng[1,2], Qixun Li[2], Muhammad Iskandar B. Salaman[1], Chaolong Wei[1], Qilun Wang[3], Xuehu Ma[2], Bin Liu[3], and Andrew Barnabas Wong[1,4,*]

[1]Department of Materials Science and Engineering, National University of Singapore, Singapore 117575, Singapore.

[2]Institute of Chemical Engineering, Dalian University of Technology, Dalian 116024, China.

[3]Department of Materials Science and Engineering, City University of Hong Kong, Hong Kong SAR 999077, China.

[4]Department of Chemical and Biomolecular Engineering, National University of Singapore, Singapore 117575, Singapore.

This PDF file includes:

Theoretical calculation of Laplace pressure generated by the microgrooves

Experimental methods

Supplementary Figure S1 to S28

Supplementary Tables S1 to S6

Supplementary References

Theoretical calculation of Laplace pressure generated by the microgrooves

Figure S1 (a) shows a schematic of the physical model used in this analysis. In this model, we assume that as the electrolyte penetrates the microgrooves, the three-phase line advances in a constant contact angle mode.[1] According to the Young-Laplace equation, the Laplace pressure generated by the liquid-gas meniscus in the microgrooves can be expressed by,

$$\Delta P_{\mathrm{L}} = \frac{2\sigma_{\mathrm{l\text{-}g}}}{R} \tag{S1}$$

where $\sigma_{\mathrm{l\text{-}g}}$ is the liquid-gas surface tension, R is the curvature radius of the liquid-gas meniscus and can be calculated through geometrical analysis,

$$R = \frac{\tan\alpha\left(h-d\right)}{\cos\left(\alpha-\theta+\pi\right)} \tag{S2}$$

where α is the half angle of the microgroove, h is the height of the nanowires, d is the intruding depth of the electrolyte into the nanowire arrays, and θ is the contact angle of the electrolyte on the nanowires.

Thus, the Laplace pressure that prevent electrolyte intrusion can be given by,

$$\Delta P_{\mathrm{L}} = 2\sigma_{\mathrm{l\text{-}g}}\frac{\cos\left(\alpha-\theta+\pi\right)}{\tan\alpha\left(h-d\right)} \tag{S3}$$

Figure S1(b) illustrates the theoretical change in Laplace pressure as the position of the liquid-gas meniscus varies within the microgrooves. The results indicate that as the electrolyte penetrates from the top to the bottom of the V-shaped microgrooves (d changes from 0 to 10 μm), the Laplace pressure rises sharply from 3.8 kPa to 76.0 kPa, demonstrating strong anti-flooding capabilities. In addition to the microgrooves, the densely packed nanowires at the top of the bundle, with a narrow pitch of approximately 100 nm, generate a Laplace pressure of around 72 kPa, further preventing electrolyte penetration. This synergistic effect between the hierarchical microgroove and nanowire structures enables NAM-30 to maintain excellent superhydrophobicity and outstanding anti-flooding performance.

In addition to the position of liquid-gas meniscus, the nanowire length dependence of Laplace pressure is also plotted, as shown in Figure S1(c). The result illustrates a dramatic

drop of the Laplace pressure as the nanowire length increases, suggesting that the ability of NAMs for resisting electrolyte intrusion is reduced.

Experimental Methods

NAMs preparation

Superhydrophobic nanowire arrays with microgrooves (NAMs) were fabricated on Cu foil using a two-step double-pass anodic alumina oxide (AAO) template-assisted electrodeposition method. The Cu foils (99.999% purity, 0.2 ×20 ×10 mm) used in the experiments were polished with 800, 1500, 2000, 3000, and 5000 mesh sandpaper in sequence. After mechanical polishing, it was electropolished in 85% phosphoric acid at 3 V against a counter electrode for 1 min. Before utilization, the Cu foil was supersonically cleaned with acetone, ethanol, and deionized water for 5 min in sequence and then dried with nitrogen gas. A schematic illustration of the custom-made setup for the first step of electrodeposition is shown in Figure S2. The plating solution was composed of copper pyrophosphate ($Cu_2P_2O_7 \cdot xH_2O$, Sigma–Aldrich), potassium pyrophosphate ($K_4P_2O_7 \cdot 3H_2O$, Sigma–Aldrich), ammonium citrate tribasic ($C_6H_{17}N_3O_7$, Sigma–Aldrich, 97%), and deionized water (Milli-Q, >18 MΩ cm) with a mass ratio of 6:25:2:100. A double-pass AAO template (Bonding Chemical) wetted with electroplating solution was placed on Cu foil. A filter paper completely wetted by the plating solution was placed on the AAO template to store the plating solution. The counter Cu foil was placed over the filter paper to form a 2-electrode system. The first step of electrodeposition was carried out at -0.8 V for 60 min. In the second step, electrodeposition was carried out in a single cell with three electrodes for another 60 min. The applied potential was -0.8 V vs. Ag/AgCl. After electroplating, the cathode was rinsed and immersed in 2 M NaOH (Sigma–Aldrich, 98%) solution for 3 hours to completely dissolve the AAO template. The Cu nanowire arrays were obtained after being washed with deionized water and dried with nitrogen gas. Finally, the hydrophobic functionalization of NAMs was achieved by immersing the as-fabricated sample in a 2.5 mM *n*-octadecanethiol ($C_{18}H_{38}S$, Sigma–Aldrich, 98%) solution (dissolved in ethanol) at 70 °C for 1 h.

Sample characterization

The morphology of the samples was observed by a field-emission transmission scanning electron microscope (FE-SEM, JSM-7610F) equipped with an energy-dispersive spectrometer (EDS, X-Max). The surface wettability was characterized using a contact angle goniometer (Dataphysics, TBU100) at room temperature (25 °C), with the probe liquid being 4 μL of deionized water. X-ray photoelectron spectroscopy (XPS) data were collected on a Kratos AXIS Ultra spectrometer. The raw spectra were calibrated with the C 1s peak at 284.8 eV, which is commonly assigned to the adventitious carbon-containing species. X-ray diffraction (XRD) patterns were recorded on a Shimadzu XRD-6000 X-ray diffractometer with a Cu Kα source and a Rigaku Miniflex 600 diffractometer.

Electrochemical measurements

The CO_2 electrochemical reduction reaction was carried out in a gas-phase-connected H-type electrochemical cell with a standard three-electrode system connected to a potentiostat (Bio-Logic, VMP-300) for data collection. Each compartment of the H-cell was filled with 40 mL electrolyte (0.1 M $KHCO_3$) and separated by an anion exchange membrane (AEM, Fumasep FAS-50). Pt foil and Ag/AgCl electrode filled with 3.5 M KCl solution were used as the counter electrode and the reference electrode, respectively. Before the CO_2RR test, CO_2 gas was bubbled into the electrolyte at a flow rate of 20 mL□min^{-1} for 20 min to saturate the electrolyte with CO_2. The gas flow rate was controlled at 20 sccm during the test. The cathodic electrodes were placed with a tilt angle of 30° such that the gas bubble flow from the bottom could impact the electrode surface. The electrode potentials were rescaled to the reversible hydrogen electrode (RHE) with *i*R compensation using the following equation,

$$E_{\mathrm{RHE}} = E_{\mathrm{Ag/AgCl}} + 0.2046 + 0.0591 \times \mathrm{pH} - I\mathrm{R} \quad \text{(S4)}$$

where I is the current and R is the internal resistance.

Cyclic voltammetry curves in hydroxyl UPD test were recorded at a scan rate of 20 mV/s in 1 M KOH solution for all NAMs.[2]

The ECSA values were estimated from double-layer capacitance (C_{dl}) currents at different scan rates ranging from 20 to 160 mV·s^{-1}. The C_{dl} values were achieved by a linear fit of the slope. The ECSA was then determined from the difference between the capacitance of the NAMs relative to that of the Cu foil with a surface area of 1 cm^2.

CO stripping voltammetry was performed immediately after 40 min of CO_2RR at -1.2 V vs. RHE. CO stripping chronoamperometry (CA) was performed by a 4-step CA sequence for double layer charging correction [3]. A schematic illustration of this procedure is also shown in Figure S20. In step I, we performed CO_2RR for 40 min at -1.2 V vs. RHE. During this step, the key intermediate CO* was produced. In step II, the potential was switched to 0.2 V vs. RHE for 3 min. In this step, the current included both electrode charging and CO oxidation until CO was depleted. In step III, the potential was switched to -1.2 V vs. RHE again and sustained for 1 s, ensuring that the cathode was charged under the same conditions as in step I. In step IV, the potential was set at 0.2 V vs. RHE for 3 min, under which the current represents the electrode charging solely because negligible CO was produced in a 1 s CO_2RR process. Thus, the difference between steps II and IV represents the net CO stripping current.

Product analysis

Gas-phase products were quantified using a gas chromatograph (GC, Shimadzu, 2014C). Liquid-phase products were detected by a ^{1}H nuclear magnetic resonance (NMR) spectrometer (Bruker, 400 MHz). The FE was calculated based on the following equation,

$$FE(\%)=\frac{n_{\text{product}}\times n_{\text{electrons}}\times F}{It} \tag{S5}$$

where n_{product} is the product measured (mol), $n_{\text{electrons}}$ is the number of transferred electrons for the generation of the product, F is the Faraday constant (C□mol^{-1}), I is the current, and t is the reaction time for the collected products.

Laser scanning confocal microscopy

The gas–liquid–solid interfaces were directly observed through a series of confocal images at different depths within the nanowire arrays. All measurements were carried out on a laser scanning confocal microscope (Leica, Stellaris 8) equipped with a ×63 water objective lens. Rhodamine B was used as the fluorescent dye for imaging the electrolyte at a concentration of 1 μg□mL^{-1}. Accordingly, a 540 nm laser was used as the excitation source. The reflection and emission signals were recorded. The confocal images were then obtained by overlaying the reflection and emission signals.

Computational modeling

To clarify the underlying mechanism by which the microscopic wetting state boosts CO_2RR performance, we carried out a three-dimensional and steady-state numerical simulation of the mass transfer process in CO_2RR to determine the distribution of pH, CO_2 concentration, and fluxes of species involved in chemical equilibria within the structure under the applied operating conditions by exploiting the finite-element method on the COMSOL Multiphysics® platform.[4]

In this model, the electroreactions of CO_2RR and HER in 0.1 M $KHCO_3$ saturated with CO_2 at −1.2 V vs. RHE; the carbonate species equilibria of OH^-, CO_2, HCO_3^-, and CO_3^{2-}; the mass diffusion of CO_2, OH^-, H^+, K^+, HCO_3^-, and CO_3^{2-}; and the local hydrolysis of K^+ in the electrolyte phase are considered.

The assumptions involved in this model are as follows: (1) The mass transfer processes are in a steady state, and diffusion occurs near the surface with negligible convection. (2) CO_2RR and HER occur at the liquid–solid interface, and the CO_2RR and HER-induced mass consumption (CO_2) and generation (OH^-) are presented. (3) Because of the Cassie-Baxter wetting state and the stable CO_2 gas layer underneath the nanowires, a constant concentration condition of saturated CO_2 is at the gas–liquid interface of NAM-30. (4) Due to the partial wetting of NAM-10, *i.e.*, gas layer is underneath the nanowires at some sites and liquid fulfills the nanowires at other sites, we carried out the two different simulations of the above two cases and used the average value for comparison among the different samples. (5) In the NAM-0 model, the electrolyte is filled with nanowires as a result of the Wenzel wetting state.

The governing equation of the mass transfer processes can be described by the Nernst−Planck equation,

$$N_i = -D_i \nabla c_i - z_i \frac{D_i}{RT} F c_i \nabla \phi_l \qquad \text{(S6)}$$

where the first and second terms represent the mass transfer induced by the concentration gradient and ion migration under the electric field, respectively. At steady state, the governing equations for the different species can be expressed by,

$$\nabla\left(-D_{CO_2} \nabla c_{CO_2}\right) = k_{1r} c_{HCO_3^-} - k_{1f} c_{CO_2} c_{OH^-} \qquad \text{(S7)}$$

$$\nabla\left(-D_{\mathrm{HCO_3^-}}\nabla c_{\mathrm{HCO_3^-}}-z_{\mathrm{HCO_3^-}}\frac{D_{\mathrm{HCO_3^-}}}{RT}Fc_{\mathrm{HCO_3^-}}\nabla\phi_1\right)= \tag{S8}$$

$$k_{1\mathrm{f}}c_{\mathrm{CO_2}}c_{\mathrm{OH^-}}-k_{1\mathrm{r}}c_{\mathrm{HCO_3^-}}+k_{2\mathrm{r}}c_{\mathrm{CO_3^{2-}}}-k_{2\mathrm{f}}c_{\mathrm{HCO_3^-}}c_{\mathrm{OH^-}}$$

$$\nabla\left(-D_{\mathrm{CO_3^{2-}}}\nabla c_{\mathrm{CO_3^{2-}}}-z_{\mathrm{CO_3^{2-}}}\frac{D_{\mathrm{CO_3^{2-}}}}{RT}Fc_{\mathrm{CO_3^{2-}}}\nabla\phi_1\right)=k_{2\mathrm{f}}c_{\mathrm{HCO_3^-}}c_{\mathrm{OH^-}}-k_{2\mathrm{r}}c_{\mathrm{CO_3^{2-}}} \tag{S9}$$

$$\nabla\left(-D_{\mathrm{OH^-}}\nabla c_{\mathrm{OH^-}}-z_{\mathrm{OH^-}}\frac{D_{\mathrm{OH^-}}}{RT}Fc_{\mathrm{OH^-}}\nabla\phi_1\right)=k_{1\mathrm{r}}c_{\mathrm{HCO_3^-}}-k_{1\mathrm{f}}c_{\mathrm{CO_2}}c_{\mathrm{OH^-}}+k_{2\mathrm{r}}c_{\mathrm{CO_3^{2-}}}-k_{2\mathrm{f}}c_{\mathrm{HCO_3^-}}c_{\mathrm{OH^-}} \tag{S10}$$

To solve the above equations, several boundary conditions are adopted, as shown in Figure S25. Bulk conditions (1), *i.e.*, a constant concentration of species, are considered at the top end of the electrolyte.

$$c_i = cons.\left(i = \mathrm{CO_2},\ \mathrm{HCO_3^-},\ \mathrm{HCO_3^{2-}},\ \mathrm{OH^-},\ \mathrm{K^+}\right) \tag{S11}$$

Symmetric condition (2), *i.e.*, zero flux of all species, are imposed at the lateral wall of the electrolyte to reflect the periodic 1/6 (60°) of the circumferential nanowire array and the symmetric V-shaped groove.

$$N_i=-D_i\nabla c_i-z_i\frac{D_i}{RT}Fc_i\nabla\phi_1=0 \tag{S12}$$

At the boundaries of the nanowire surface (3), zero-flux conditions are adopted for nonreactive species,

$$N_i=-D_i\nabla c_i-z_i\frac{D_i}{RT}Fc_i\nabla\phi_1=0\ (i=\mathrm{HCO_3^-},\ \mathrm{HCO_3^{2-}},\ \mathrm{K^+}) \tag{S13}$$

while reaction conditions are imposed for CO_2 and OH^-, which are involved in CO_2RR and HER, respectively. The reactions occurring at the solid–liquid interface can be determined by the Nernst equation and the applied electrode potential,

$$E_{\mathrm{eq}}=E^0_{\mathrm{eq}}-\frac{RT}{nF}\ln\left(\frac{c^n_{\mathrm{OH^-}}}{c^m_{\mathrm{CO_2}}}\right) \tag{S14}$$

For the CO_2RR, a first-order dependence of the CO_2 consumption rate on the concentration is assumed. Consequently, the boundary condition for consumption of CO_2 at the solid–liquid interface can be expressed by,

$$N_i=-D_{\mathrm{CO_2}}\nabla c_{\mathrm{CO_2}}=k_{\mathrm{CO_2}}c_{\mathrm{CO_2}} \tag{S15}$$

The rate constant k_{CO2} can be obtained by coupling the electron transfer (partial current density of products) and the mass conservation (FE of products) acquired from the experimental results,

$$\left(\frac{j}{F}\right)\sum_{k}\frac{\mathrm{FE}_k \cdot m_k}{z_k} = \iiint_{l-\mathrm{s\ interface}} k_{\mathrm{CO_2}} c_{\mathrm{CO_2}} dxdydz \tag{S16}$$

A zero-order dependence of the OH^- generation rate on the concentration is assumed. Similarly, the boundary condition for OH^- can be obtained by,

$$N_i = -D_{\mathrm{OH^-}} \nabla c_{\mathrm{OH^-}} = k_{\mathrm{OH^-,CO_2RR}} c_{\mathrm{CO_2}} + k_{\mathrm{OH^-,HER}} \tag{S17}$$

The rate constant k_{OH} can be obtained by coupling electron transfer (partial current density of products) and mass conservation (FE of products),

$$\left(\frac{j}{F}\right)\sum_{k}\frac{\mathrm{FE}_k \cdot m_k}{z_k} = \iiint_{l-\mathrm{s\ interface}} k_{\mathrm{OH^-,CO_2RR}} c_{\mathrm{CO_2}} dxdydz \tag{S18}$$

$$\left(\frac{j}{F}\right)\frac{\mathrm{FE}_{\mathrm{H_2}} \cdot m_{\mathrm{H_2}}}{z_{\mathrm{H_2}}} = \iiint_{l-\mathrm{s\ interface}} k_{\mathrm{OH^-,HER}} dxdydz \tag{S19}$$

The buffer effect of K^+ near the surface due to the electric potential can be described by,[5-6]

$$\left[\mathrm{K^+(H_2O)_n}\right] + \mathrm{H_2O} \leftrightarrow \left[\mathrm{KOH(H_2O)_{n-1}}\right] + \mathrm{H_3O^+} \tag{S20}$$

The reactions toward different products detected in experiments and corresponding stoichiometric parameters are given in Table S3. The equilibria involving the protonation/deprotonation of species in solution, the corresponding forward and backward reaction rate constants, and the reaction equilibrium constants are listed in Tables S4 and S5, respectively. The diffusion coefficients and bulk concentrations (initial concentrations) for the involved species in the model are listed in Table S6.

The set of above equations with boundary conditions forms a closed system for the calculation of the concentrations of all the species involved in the three-dimensional system.

Supplementary Figures

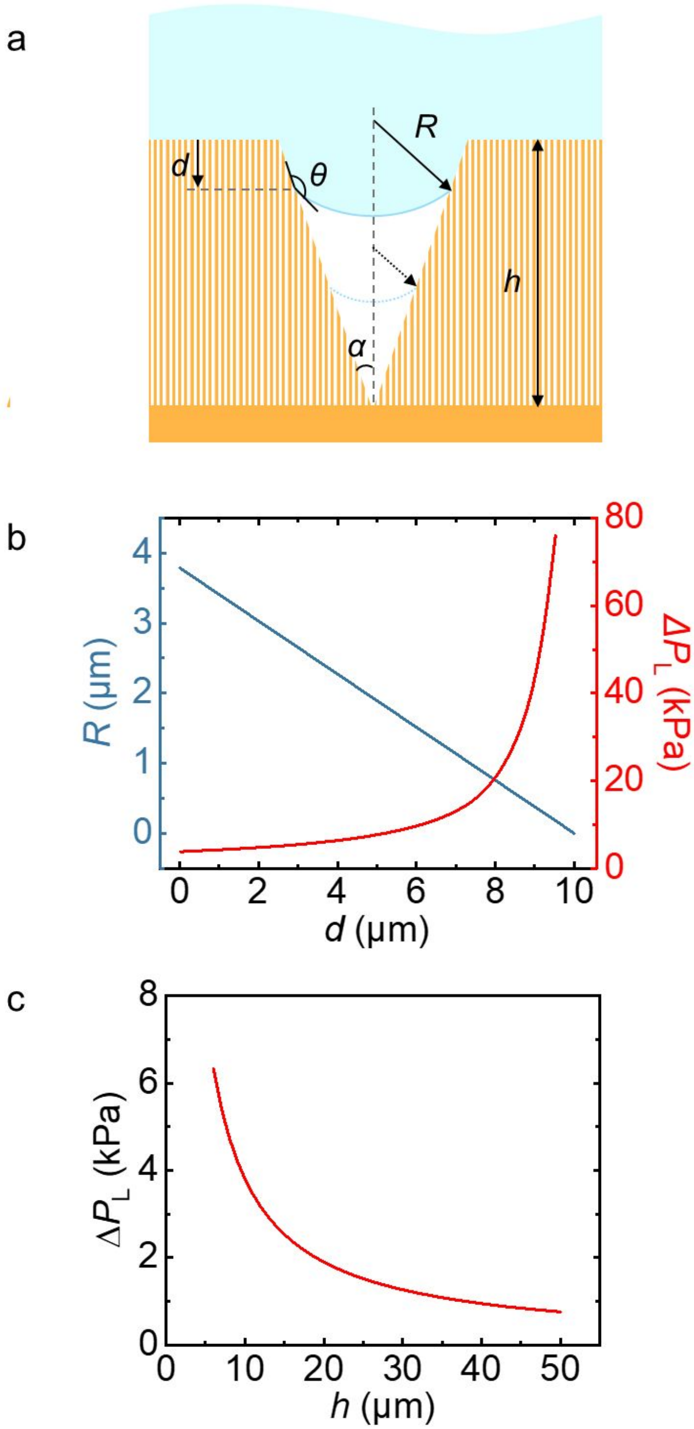

Figure S1. Mechanism of microgrooves in NAM-30 for sustaining superhydrophobicity. (a) Schematic representation of the physical model. (b) Theoretical changes in Laplace pressure with varying positions of the liquid-gas meniscus. (c) Theoretical changes in Laplace pressure with varying heights of nanowires.

a

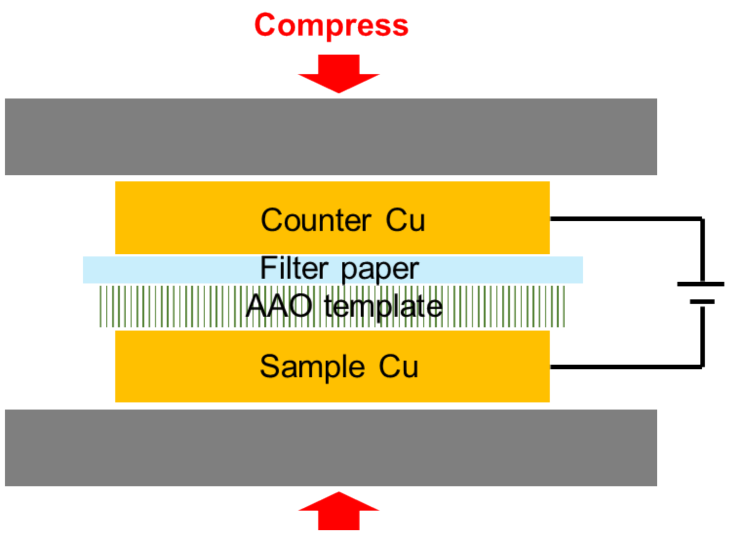

b

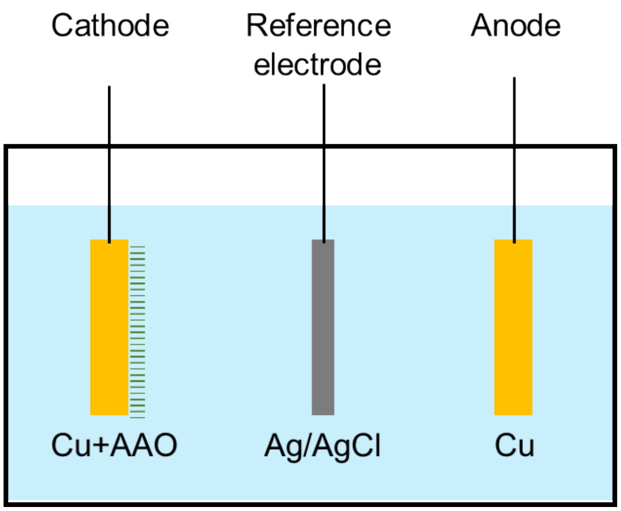

Figure S2. The configuration of the electrodeposition. (a) The configuration of the first step electrodeposition. The filter paper and AAO template are saturated with an electroplating solution. (b) The configuration of the second step electrodeposition.

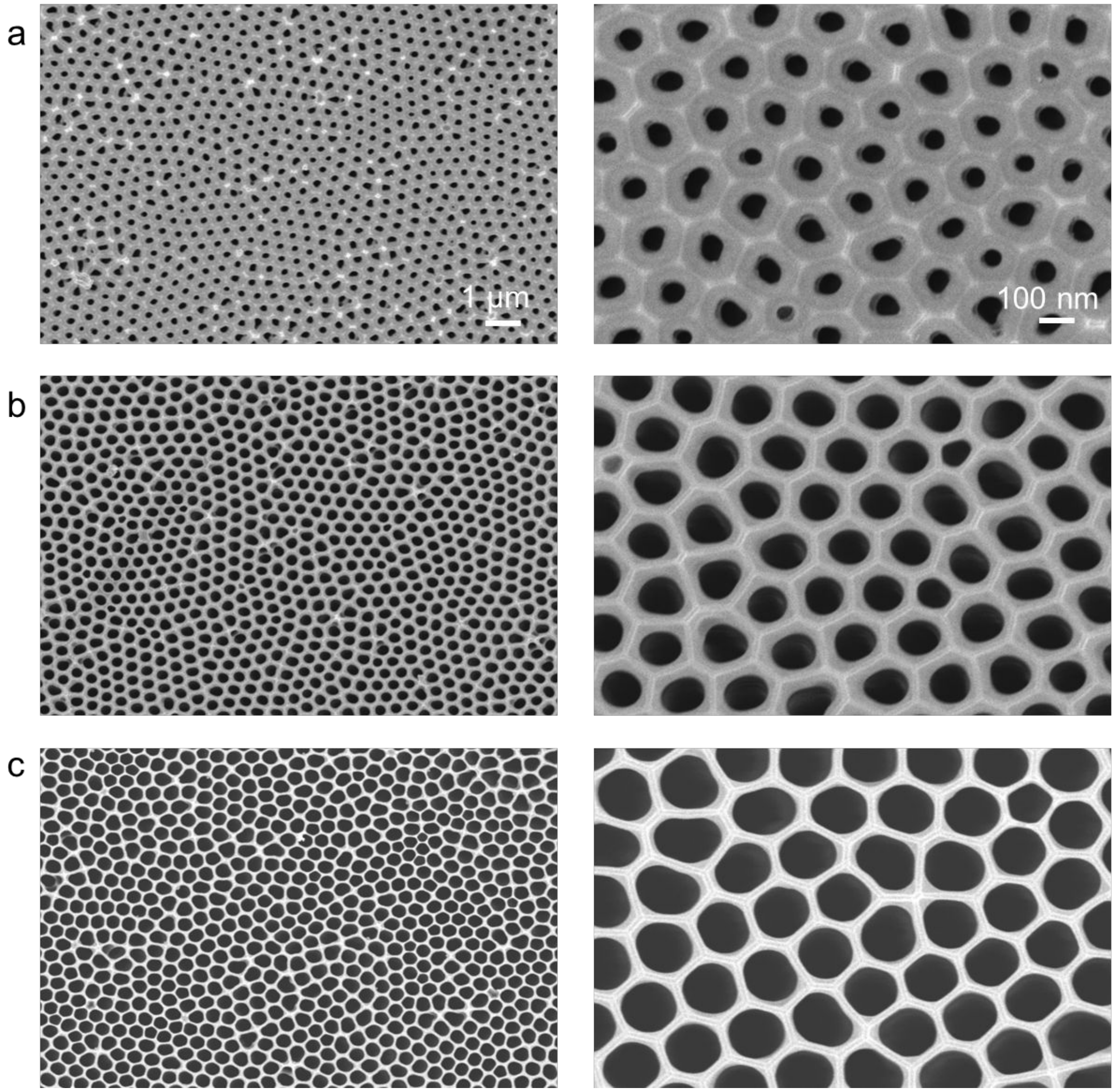

Figure S3. SEM images of AAO templates. The AAO templates are with the same pore pitch of 450 nm and different pore diameters of (a) 200 nm, (b) 300 nm, and (c) 400 nm.

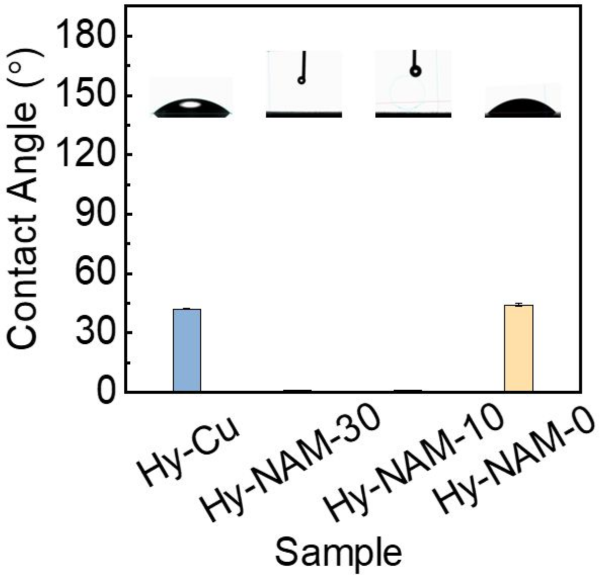

Figure S4. The contact angle measurement of the Hy-NAMs and Hy-Cu foil before hydrophobic treatment.

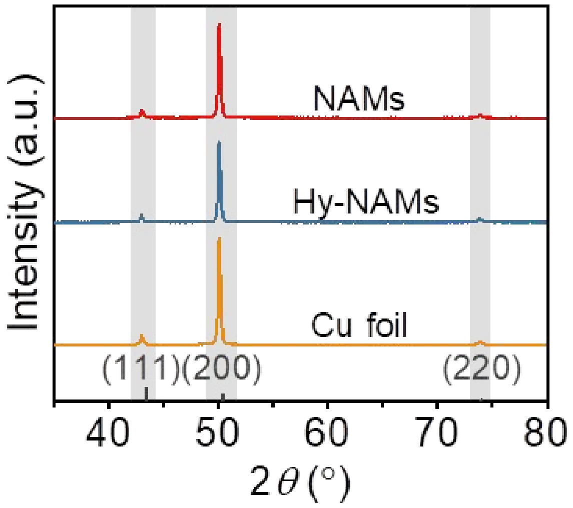

Figure S5. X-ray diffraction of the samples.

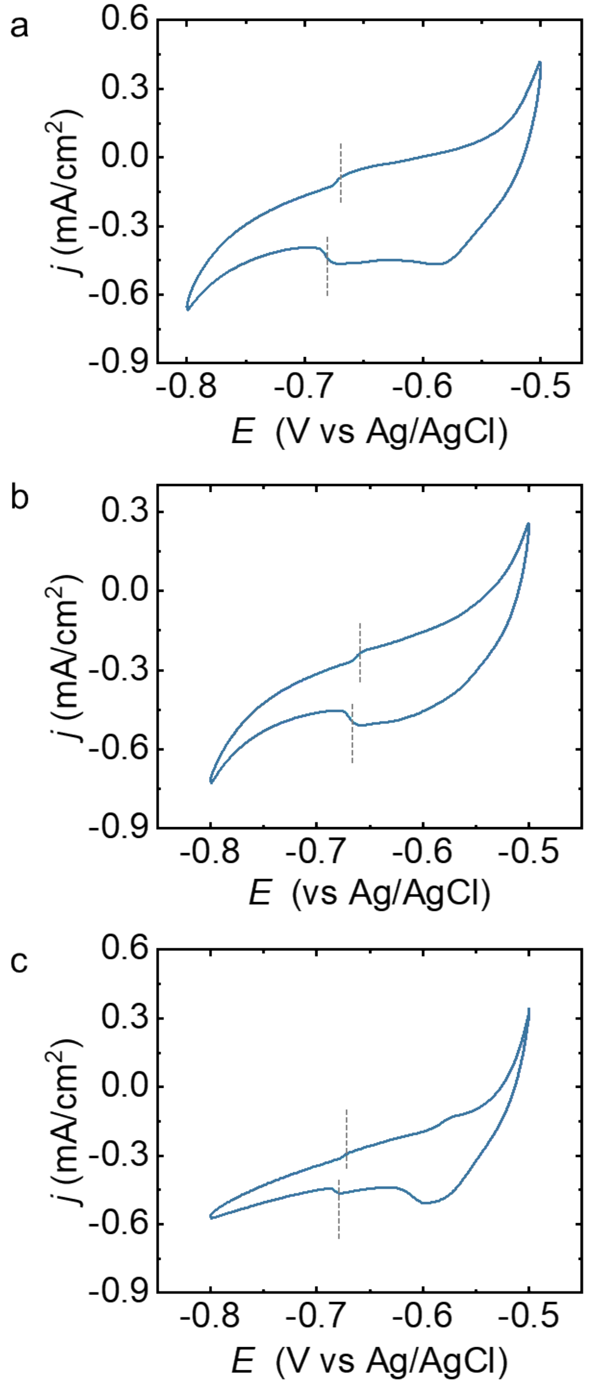

Figure S6. Potentiodynamic charging curves for (a) NAM-30, (b) NAM-10, and (c) NAM-0 before CO_2RR with a scan rate of 20 mV/s.

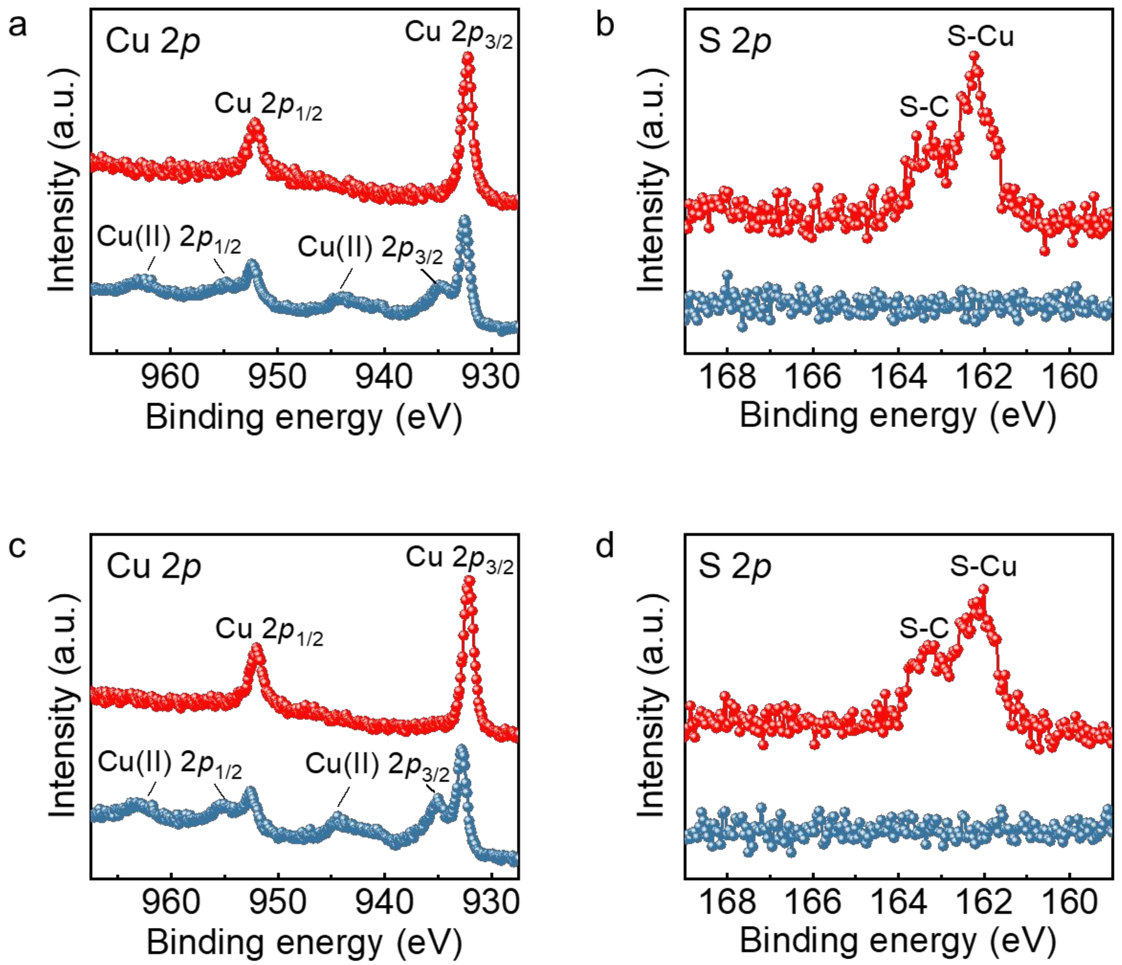

Figure S7. High-resolution XPS Cu 2*p* spectra and S 2*p* spectra of (a-b) NAM-10 and (c-d) NAM-0 before (blue) and after (red) hydrophobic treatment.

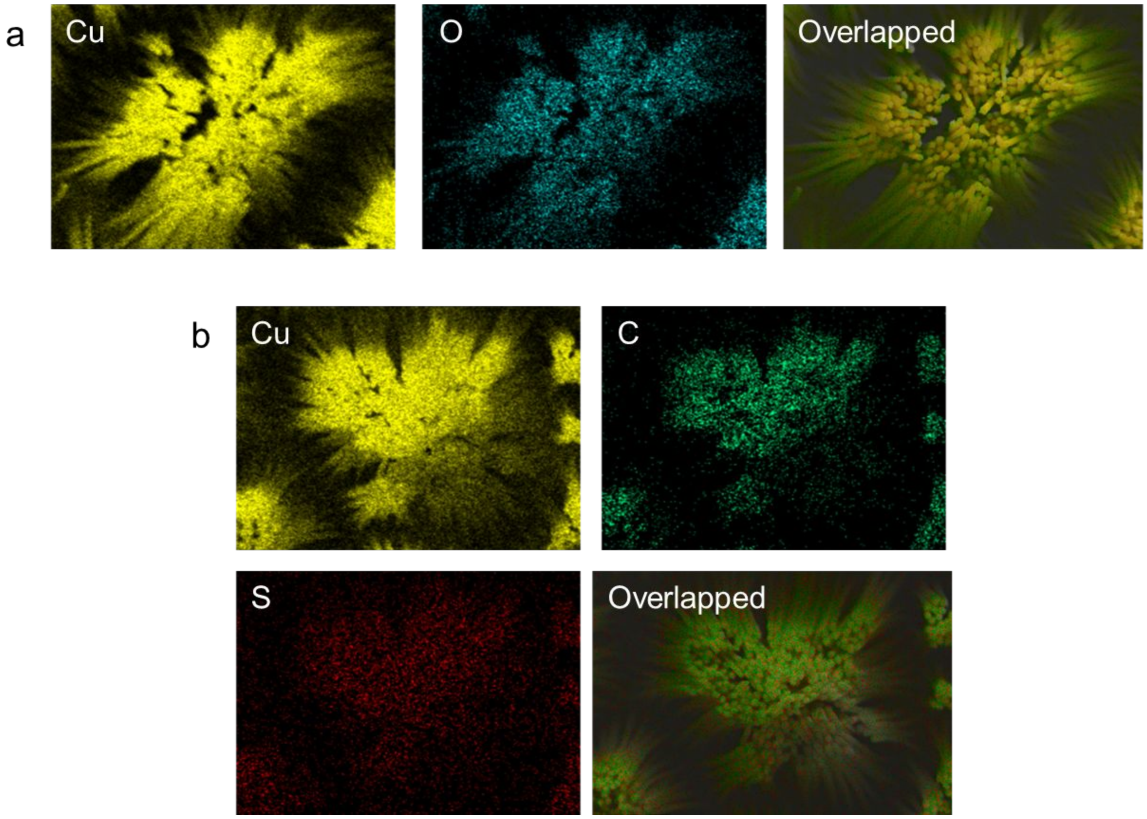

Figure S8. Energy-dispersive X-ray (EDX) mapping of (a) NAM-30 and (b) Hy-NAM-30.

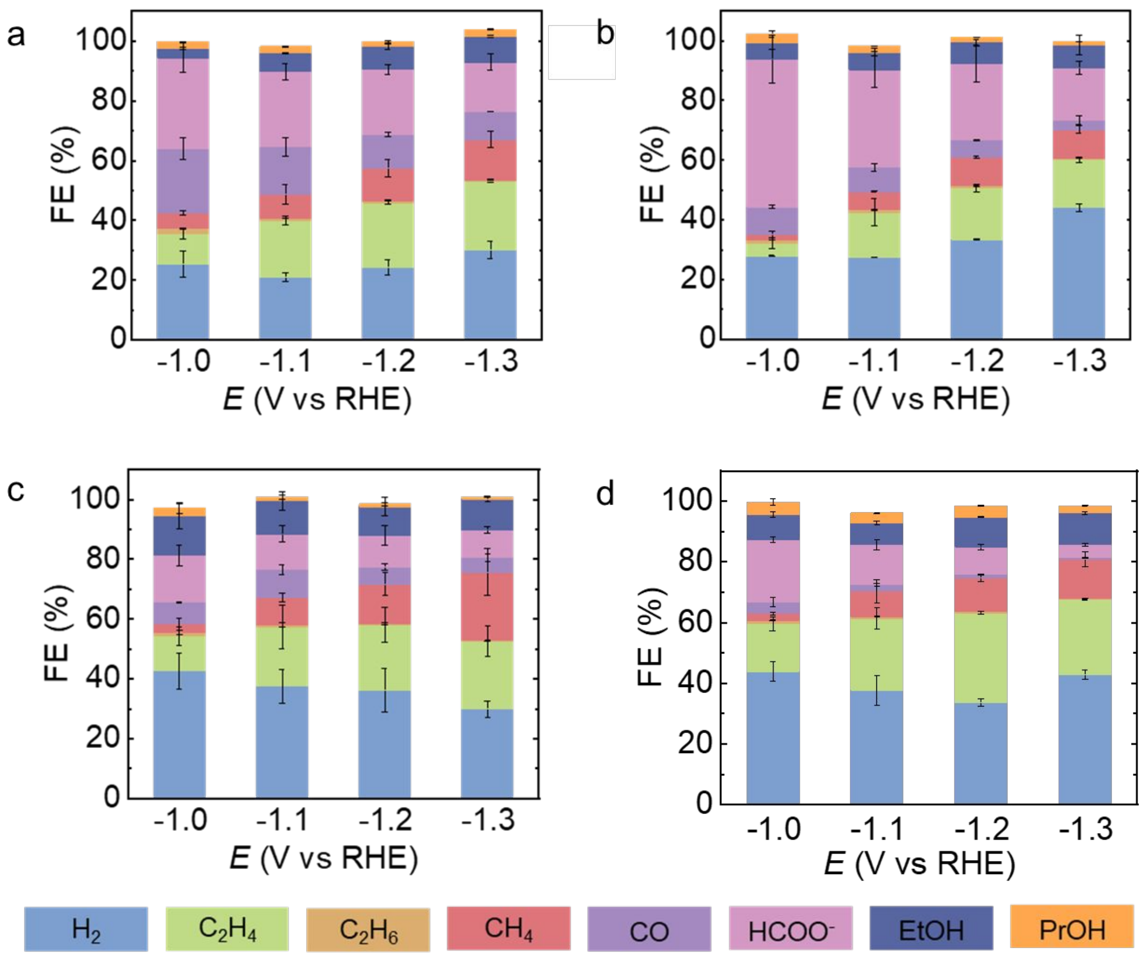

Figure S9. Selectivity of (a) NAM-10, (b) NAM-0, (c) Cu foil, and (d) Hy-NAM-30 for CO_2 electroreduction reaction in CO_2-saturated 0.1 M $KHCO_3$ electrolyte.

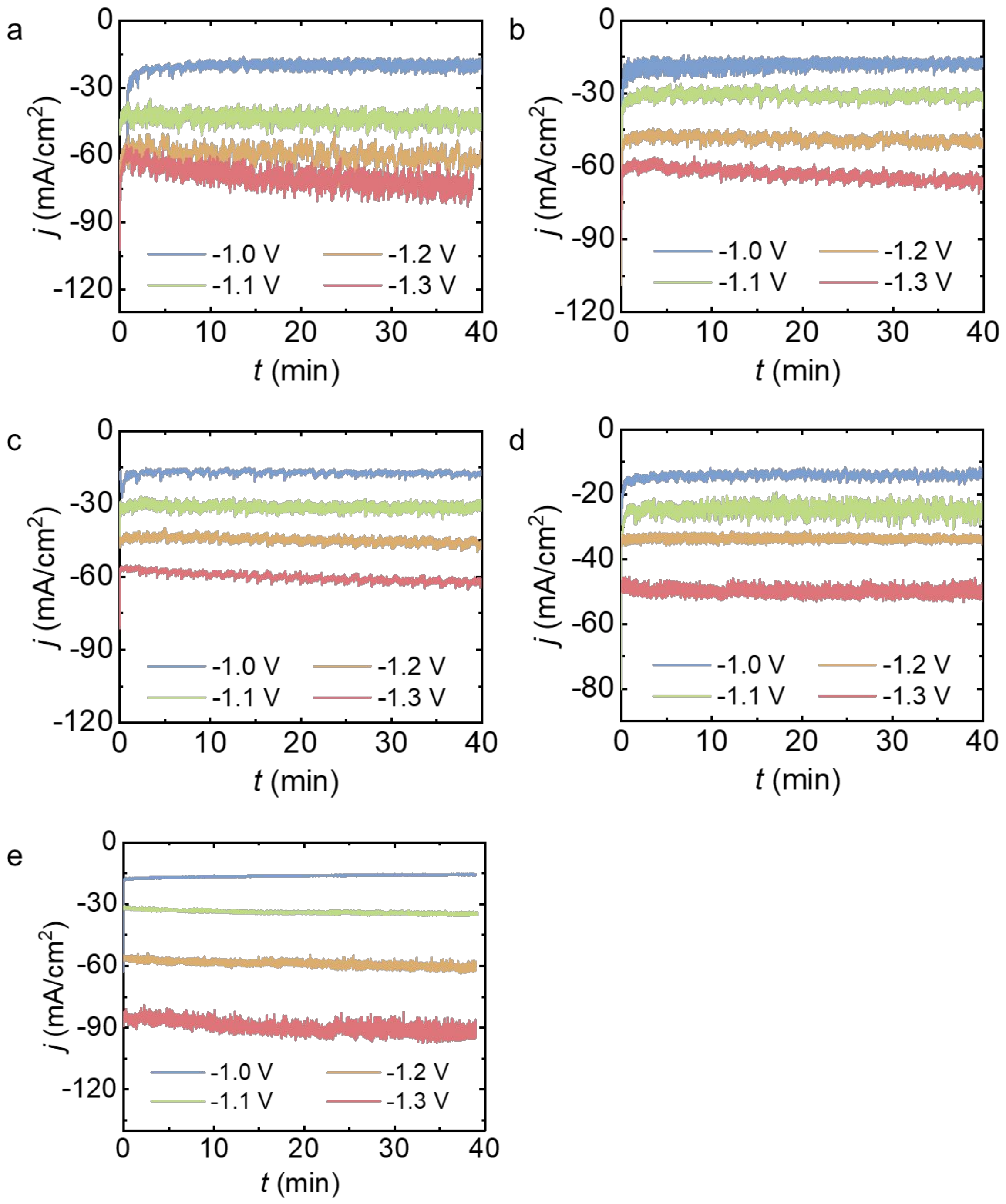

Figure S10. Current densities for (a) NAM-30, (b) NAM-10, (c) NAM-0, (d) Cu foil, and (e) Hy-NAM-30 at various potentials (vs. RHE).

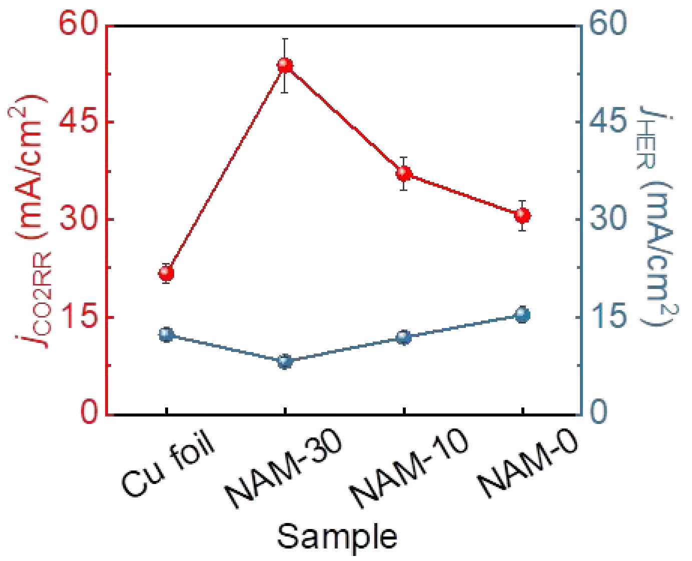

Figure S11. The geometric area normalized partial current density for different samples.

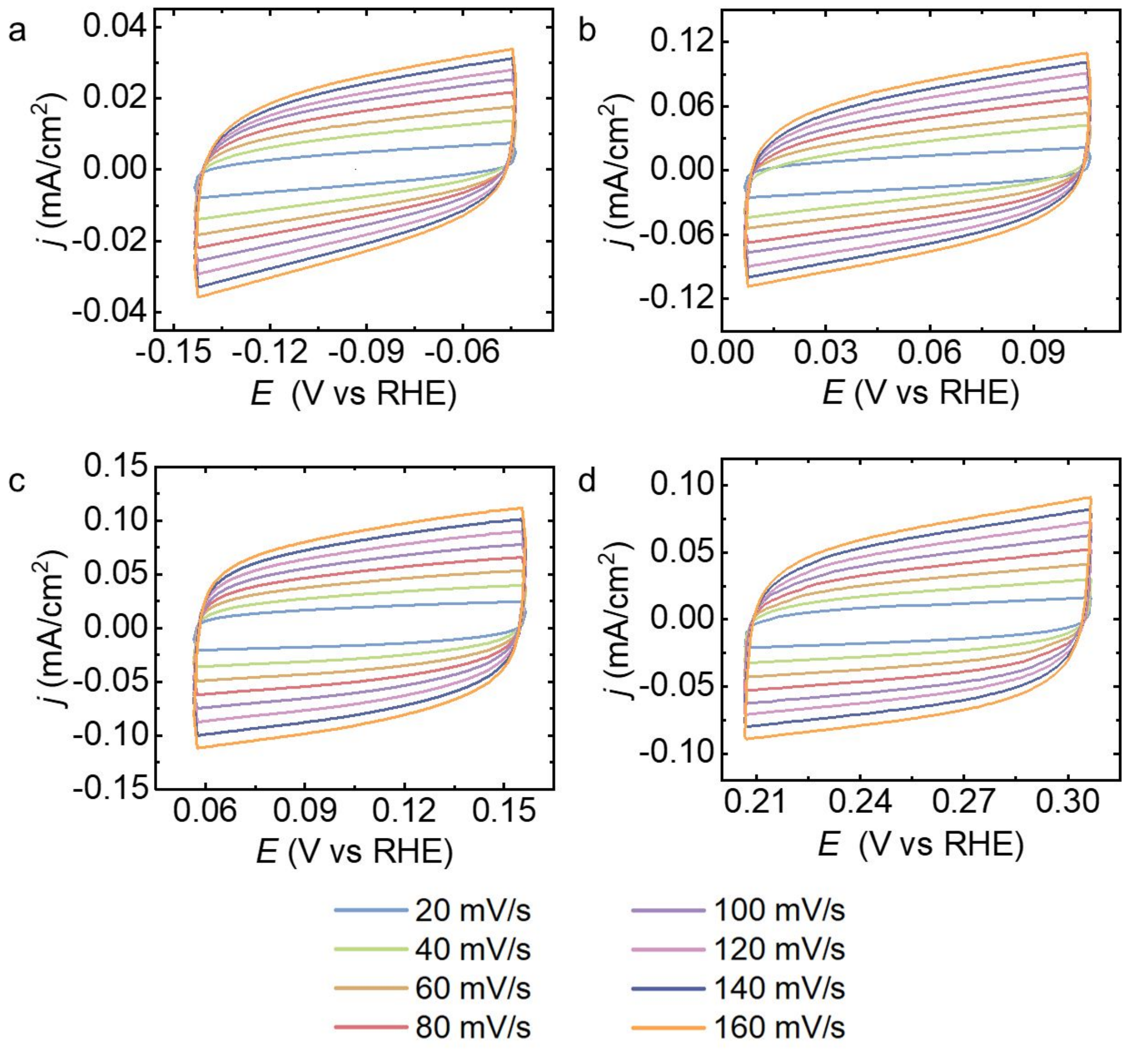

Figure S12. Cyclic voltammetry (CV) curves with various scan rates for (a) NAM-30, (b) NAM-10, (c) NAM-0, and (d) Cu foil.

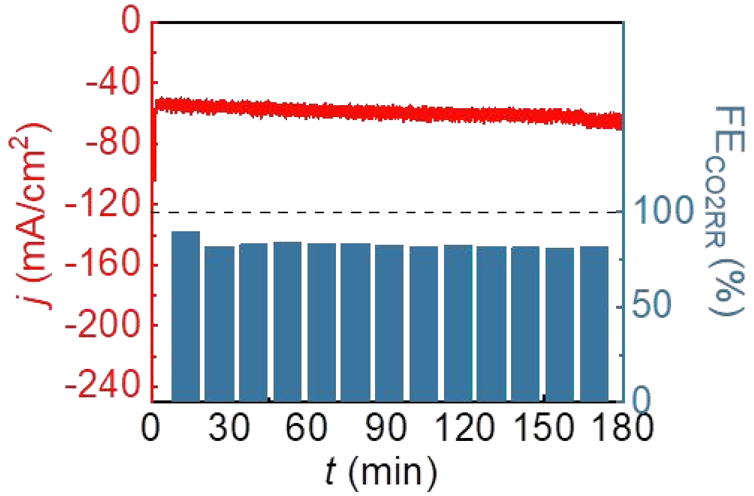

Figure S13. Stability test of the NAM-30. The current density and the FE of CO_2RR show good stability in a 180 minute test of CO_2RR.

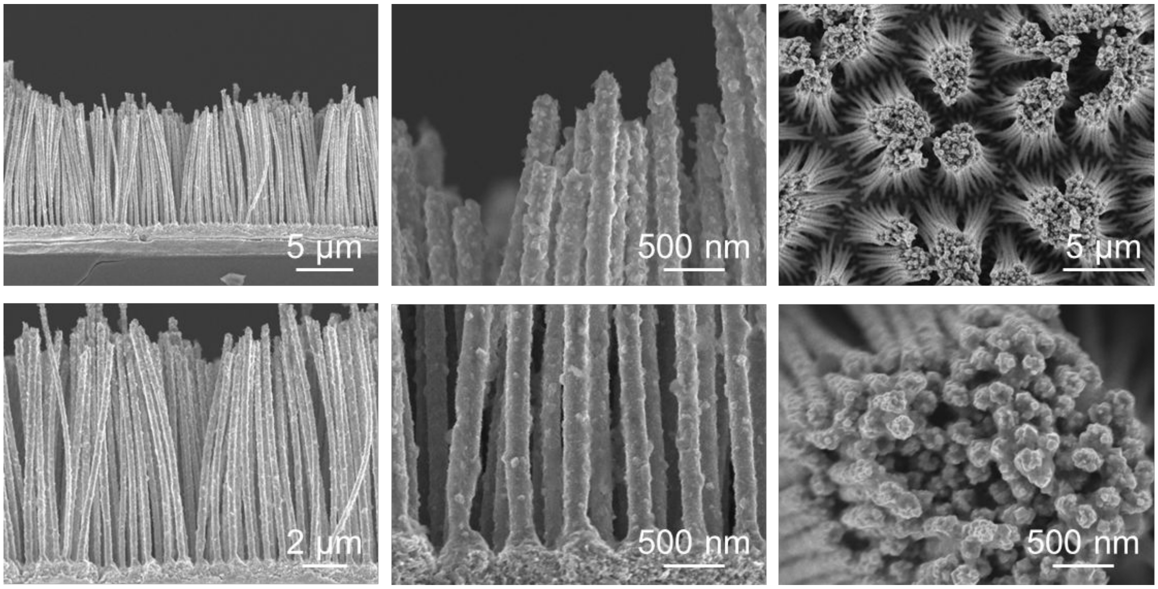

Figure S14. Structural morphology of NAM-30 after CO_2RR.

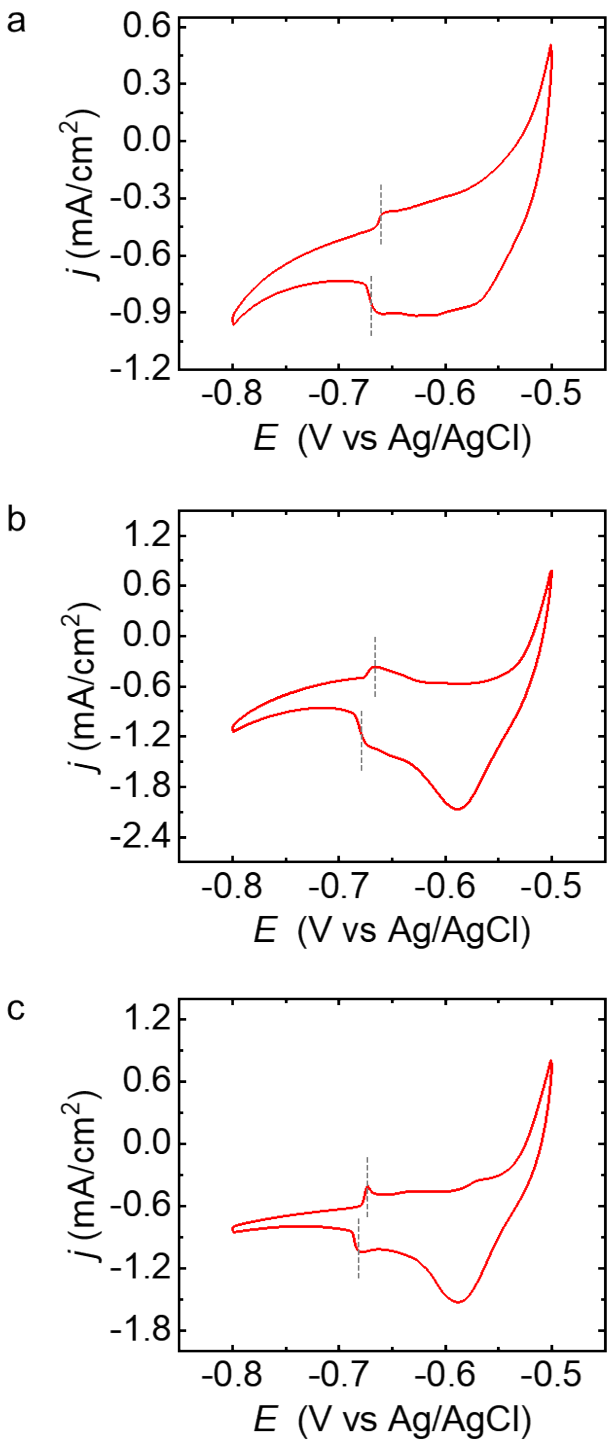

Figure S15. Potentiodynamic charging curves for (a) NAM-30, (b) NAM-10, and (c) NAM-0 after CO_2RR with a scan rate of 20 mV/s.

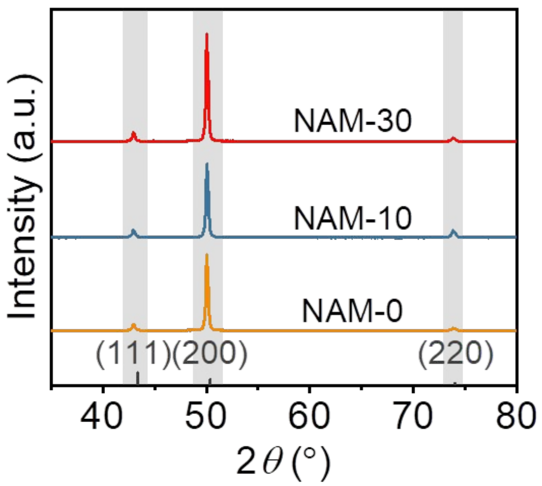

Figure S16. X-ray diffraction of the NAMs after CO_2RR.

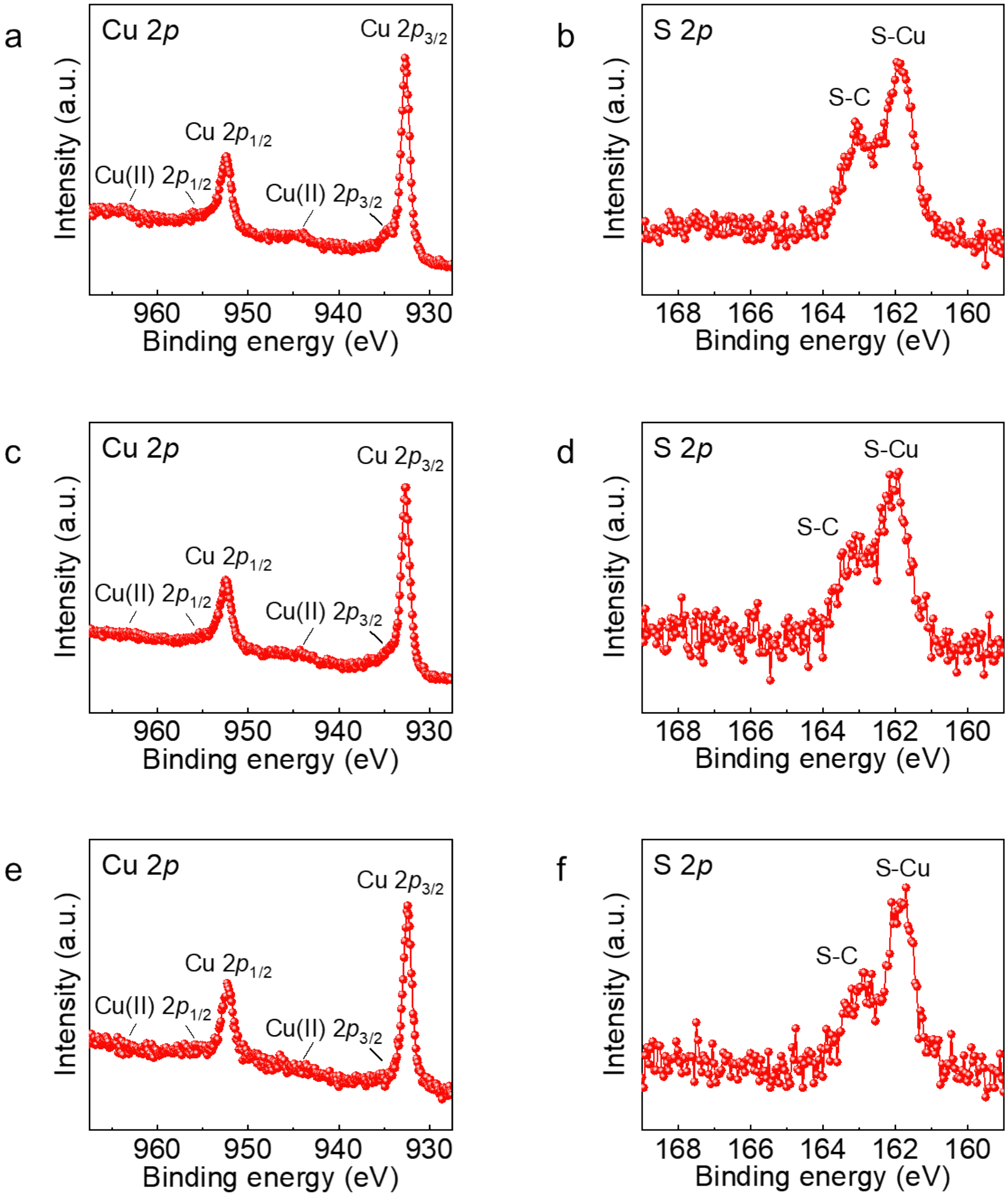

Figure S17. High-resolution XPS Cu 2*p* spectra and S 2*p* spectra of (a-b) NAM-30, (c-d) NAM-10, (e-f) NAM-0 after CO_2RR.

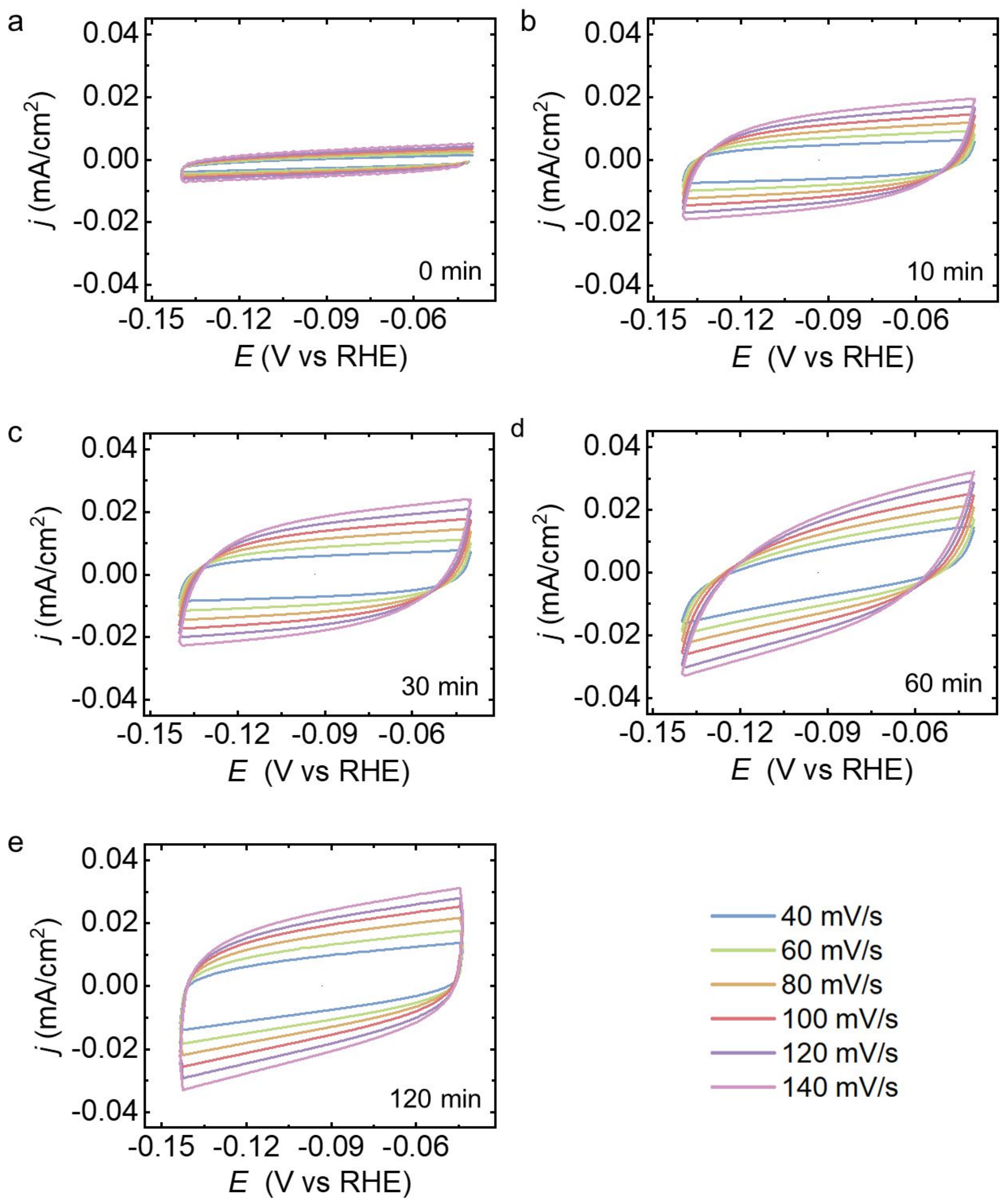

Figure S18. The cyclic voltammetry curves with various scan rates of NAM-30 at different stages of CO_2RR. (a) 0 min, (b) 10 min, (c) 30 min, (d) 60 min, (e) 120 min.

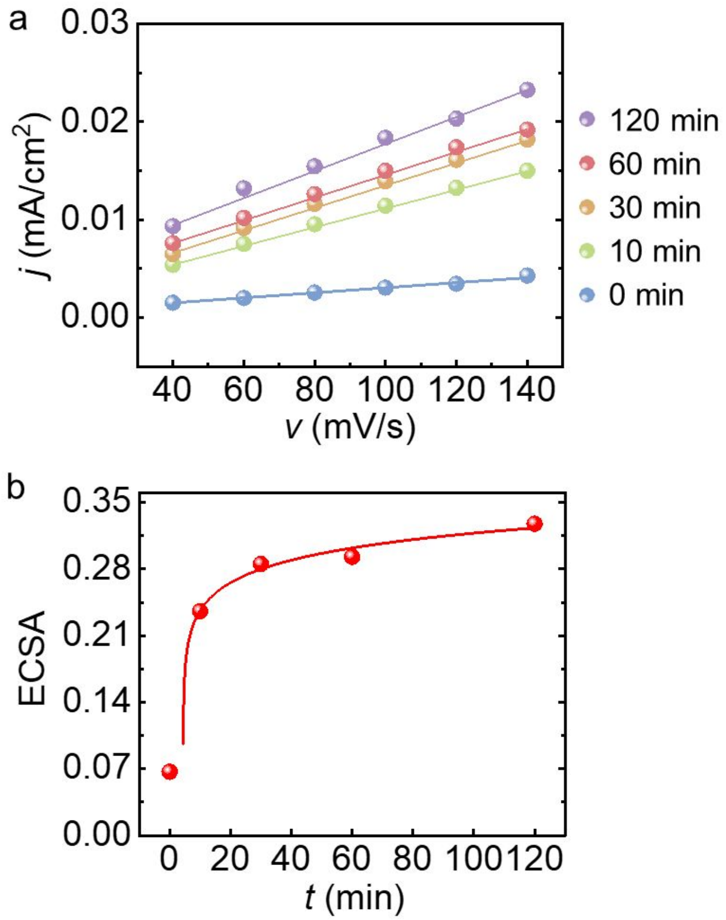

Figure S19. Evolution of (a) the double layer capacitance and (b) ECSA of NAM-30 over time, derived from the corresponding cyclic voltammetry curves in Figure S16. The line is fitted to the experimental data using a logarithmic function.

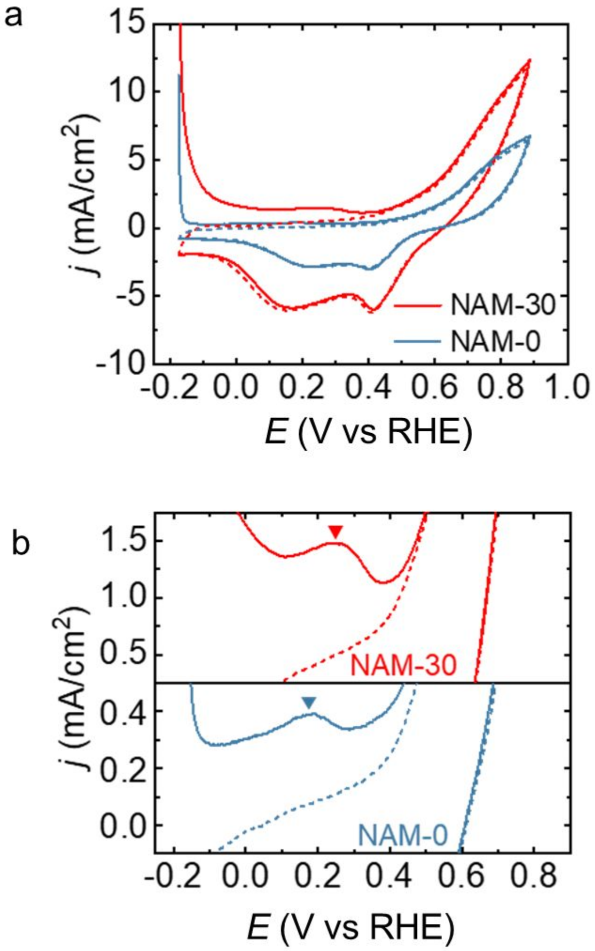

Figure S20. (a) CO stripping voltammetry of NAM-30 and NAM-0. (b) The zoomed-in CO oxidation peaks of (a), which are indicated by triangular markers. CO oxidation (line) and subsequent cycle (dashed) are obtained at a scan rate of 50 mV/s. The CO stripping was performed immediately after a 40 min operation of CO_2RR at -1.2 V vs RHE for each sample.

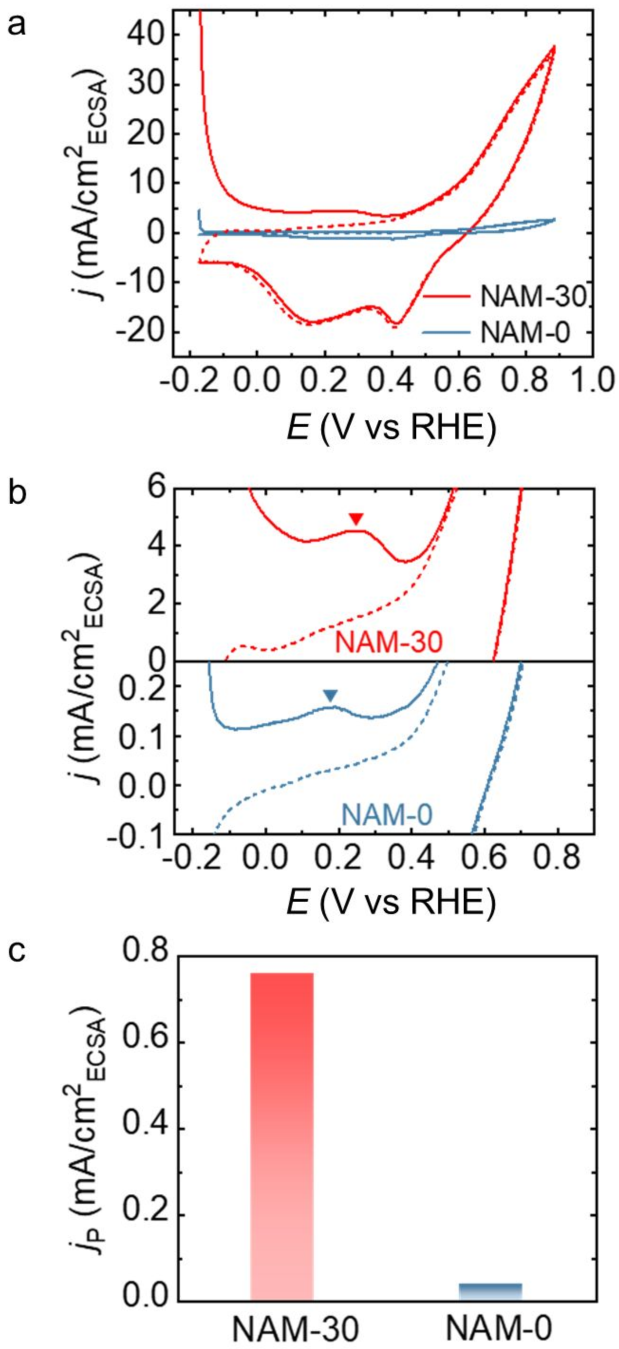

Figure S21. (a) CO stripping voltammetry normalized by ECSA of NAM-30 and NAM-0. (b) The zoomed-in CO oxidation peaks of (a), which are indicated by triangular markers. CO oxidation (line) and subsequent cycle (dashed) are obtained at a scan rate of 50 mV/s. The CO stripping was performed immediately after a 40 min operation of CO_2RR at -1.2 V vs RHE for each sample. (c) Comparison of the peak current density of CO oxidation (j_P) between NAM-30 and NAM-0.

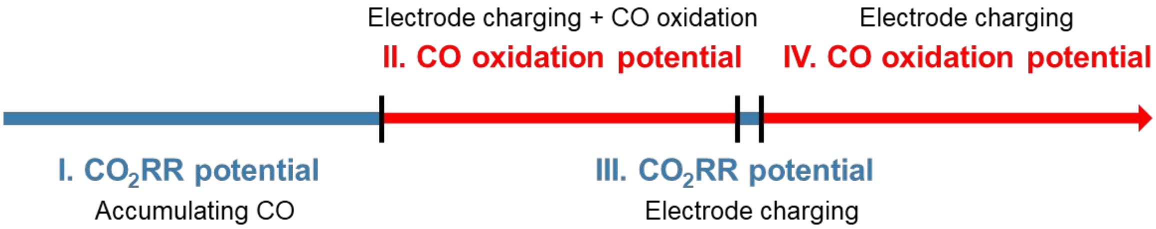

Figure S22. Schematic illustration of the double layer charging correction method for chronoamperometric CO stripping. Step I (40 min): Set potential at -1.2 V vs RHE to perform CO_2RR for producing and accumulating CO at the cathode. Step II (3 min): Change potential to 0.2 V vs RHE immediately, under which potential the current represents both the electrode charging and CO oxidation, until the CO is depleted. Step III (1 s): Change potential to -1.2 V vs RHE immediately, ensuring the cathode is charged and in the same condition as in step I. Step IV (3 min): Change potential to 0.2 V vs RHE immediately, under which potential the current represents the electrode charging solely as negligible CO is produced in a 1 s CO_2RR process. The difference between step II and IV represents the net CO stripping current.

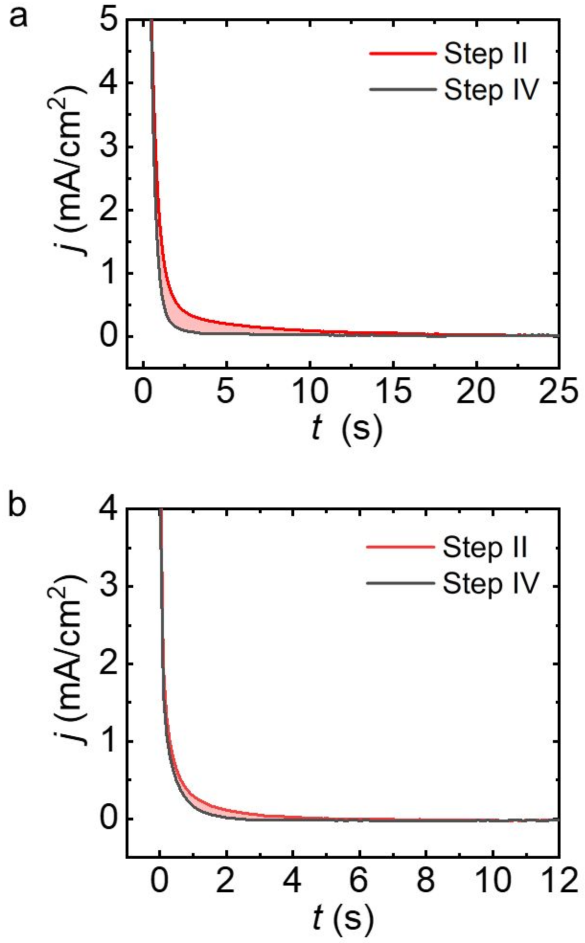

Figure S23. Chronoamperometric CO stripping at 0.2 V vs RHE for (a) NAM-30 and (b) NAM-0. The shaded area represents the net CO stripping current.

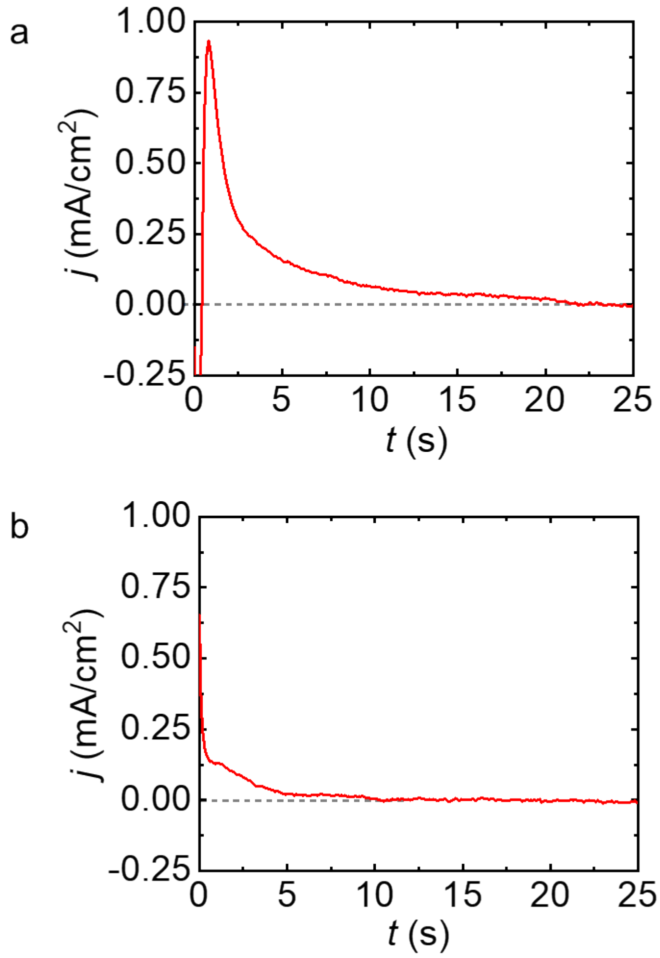

Figure S24. Net CO stripping current density after double layer correction for (a) NAM-30 and (b) NAM-0 derived from Figure S23.

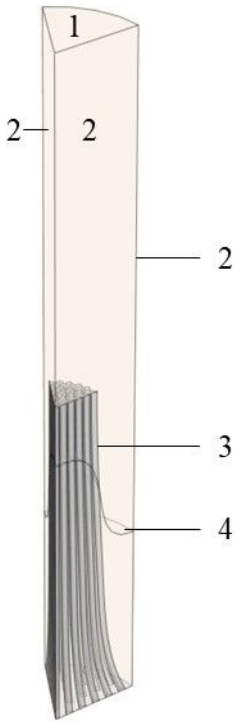

Figure S25. Boundary conditions illustration. 1-bulk condition, 2-symmetric condition, 3-reaction condition, 4-constant concentration of CO_2 if a gas layer is underneath the nanowires; if there is no gas layer, the bottom liquid–solid interface is the same with boundary 3.

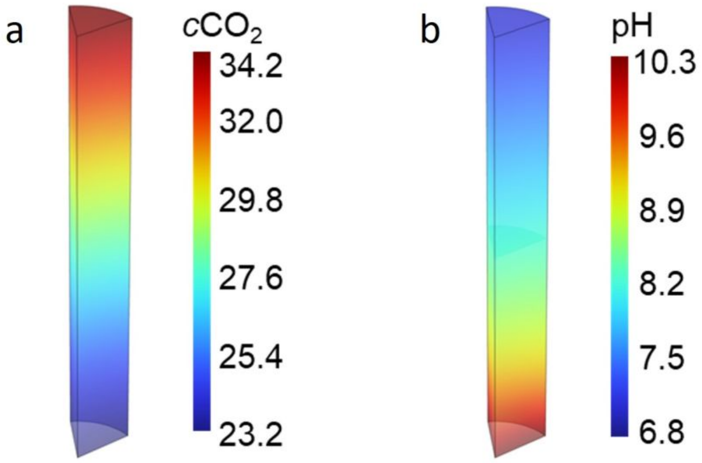

Figure S26. Mass transfer of Cu foil. (a) Contour maps of the concentration profile of CO_2. (b) Contour maps of pH.

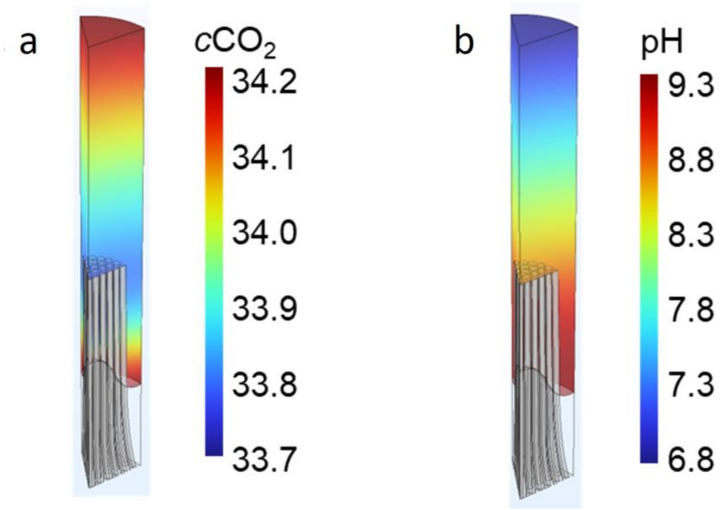

Figure S27. Mass transfer of NAM-10 with a gas layer underneath the nanowires. (a) Contour maps of the concentration profile of CO_2. (b) Contour maps of pH.

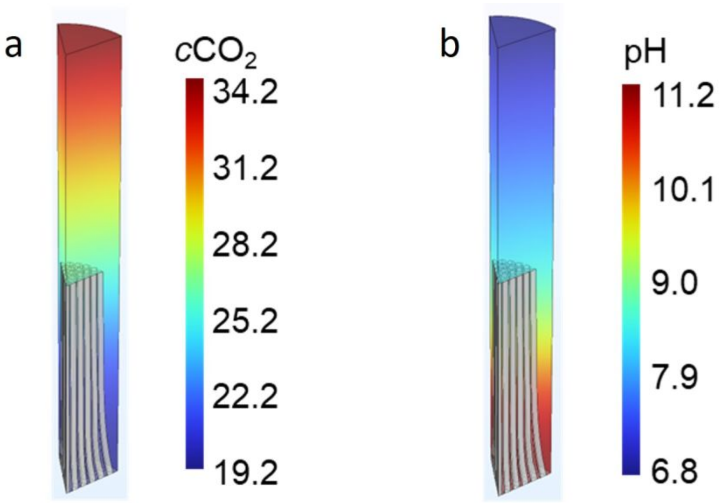

Figure S28. Mass transfer of NAM-10 in a completely Wenzel-wetting state. (a) Contour maps of the concentration profile of CO_2. (b) Contour maps of pH.

Supplementary Tables

Table S1. A summary of reported performance data of Cu nanowire catalysts.

Cu nanowire catalyst	Reported facet type	Reactor and potential (vs RHE)	Selectivity of C_{2+} products	Reference
NAMs	Cu(100)	H-cell, -1.2 V	55.7%	This work
Nanowire network	Cu(100)	H-cell, -1.0 V	15%	7
Nanowire network	Cu(200)	H-cell, -1.5 V	22%	8
Sparse nanowire	Cu(111)	H-cell, -0.93 V	18%	9
Sparse nanowires	Cu(111)	Flow cell, -0.8 V	34.1%	10
Dense nanowire arrays	Cu(111)	H-cell, -0.5 V	10%	11
Nanowire network with defects	Cu(111)	Flow cell, -1.077 V	79%	12
Nanowire with surface steps	Cu(511)	H-cell, -1.0 V	77.4%	13
Nanowire with surface steps	Cu(311)	H-cell, -1.1 V	57%	14
Nanowire network	5-fold-twinned structure, dominant Cu(100)[15]	H-cell, -1.25 V	11%	16
Nanowire network	Polycrystalline facets	H-cell, -0.8 V	3%	17
Dense nanowire arrays	Polycrystalline facets	H-cell, -1.0 V	30%	18
Dense nanowire arrays	Polycrystalline facets	H-cell, -0.495 V	2.8%	19
Dense nanowire arrays	Polycrystalline facets	H-cell, -0.8 V	25%	20
Sparse nanowire	-	H-cell, -1.1 V	34%	21

Table S2. Calculated C_{dl} and ECSA for NAMs.

Sample	C_{dl} (mF/cm^2)	ECSA
Cu foil	0.403	1
NAM-30	0.132	0.327
NAM-10	0.456	1.132
NAM-0	1.000	2.481

Table S3. Reactions considered in the model and corresponding stoichiometric parameters.

Reaction k	CO_2 (m)	OH^- (n)	e^- (z)
$2\ H_2O + 2\ e^- \rightarrow H_2 + 2\ OH^-$	0	2	2
$CO_2 + H_2O + 2\ e^- \rightarrow CO + 2\ OH^-$	1	2	2
$CO_2 + H_2O + 2\ e^- \rightarrow HCOO^- + OH^-$	1	1	2
$CO_2 + 6\ H_2O + 8\ e^- \rightarrow CH_4 + 8\ OH^-$	1	8	8
$2\ CO_2 + 8\ H_2O + 12\ e^- \rightarrow C_2H_4 + 12\ OH^-$	2	12	12
$2\ CO_2 + 10\ H_2O + 14\ e^- \rightarrow C_2H_6 + 14\ OH^-$	2	14	1
$2\ CO_2 + 9\ H_2O + 12\ e^- \rightarrow C_2H_5OH + 12\ OH^-$	2	12	12
$3\ CO_2 + 13\ H_2O + 18\ e^- \rightarrow C_3H_7OH + 18\ OH^-$	3	18	18

Table S4. Equilibria considered in the model and corresponding rate constants.

Equilibria	k_f (m^3□mol^{-1}□s^{-1})	k_b (s^{-1})
CO_2 (aq) + OH^- ↔ HCO_3^-	5.93	1.34×10^{-4}
HCO_3^- + OH^- ↔ CO_3^{2-} + H_2O	10^6	2.5×10^4

Table S5. Equilibria considered in the model and corresponding equilibrium constants.

Equilibria	K_w
$H_2O \leftrightarrow OH^- + H^+$	10^{-8}
$\left[K^+(H_2O)_n\right] + H_2O \leftrightarrow \left[KOH(H_2O)_{n-1}\right] + H_3O^+$	$10^{-8.5}$

Table S6. Diffusion coefficients at 25 °C and bulk concentrations in CO_2-saturated 0.1 M $KHCO_3$.

Species	D ($m^2 \cdot s^{-1}$)	c ($mol \cdot m^{-3}$)
CO_2	1.91×10^{-9}	34.2
HCO_3^-	9.23×10^{-10}	99.7
CO_3^{2-}	1.19×10^{-9}	29.4
OH^-	5.27×10^{-9}	6.31×10^{-5}
K^+	2.056×10^{-9}	99.7
H^+	5.27×10^{-9}	1.59×10^{-4}

Supplementary References

(1) Cheng, Y.; Liu, Y.; Ye, X.; Liu, M.; Du, B.; Jin, Y.; Wen, R.; Lan, Z.; Wang, Z.; Ma, X., Macrotextures-enabled self-propelling of large condensate droplets. *Chem. Eng. J.* **2021,** *405*, 126901.

(2) Droog, J. M.; Schlenter, B., Oxygen electrosorption on copper single crystal electrodes in sodium hydroxide solution. *J. Electroanal. Chem.* **1980,** *112*, 387-390.

(3) Arenz, M.; Mayrhofer, K. J. J.; Stamenkovic, V.; Blizanac, B. B.; Tomoyuki, T.; Ross, P. N.; Markovic, N. M., The effect of the particle size on the kinetics of CO electrooxidation on high surface area Pt catalysts. *J. Am. Chem. Soc.* **2005,** *127*, 6819-6829.

(4) Veenstra, F. L. P.; Ackerl, N.; Martín, A. J.; Pérez-Ramírez, J., Laser-microstructured copper reveals selectivity patterns in the electrocatalytic reduction of CO_2. *Chem* **2020,** *6* (7), 1707-1722.

(5) Singh, M. R.; Kwon, Y.; Lum, Y.; Ager III, J. W.; Bell, A. T., Hydrolysis of electrolyte cations enhances the electrochemical reduction of CO_2 over Ag and Cu. *J. Am. Chem. Soc.* **2016,** *138* (39), 13006-13012.

(6) Resasco, J.; Lum, Y.; Clark, E.; Zeledon, J. Z.; Bell, A. T., Effects of anion identity and concentration on electrochemical reduction of CO_2. *ChemElectroChem* **2018,** *5* (7), 1064-1072.

(7) Conte, A.; Rosati, A.; Fantin, M.; Aliprandi, A.; Baron, M.; Bonacchi, S.; Antonello, S., Advanced morphological control over Cu nanowires through a design of experiments approach. *Mater. Adv.* **2024,** *5* (22), 8836-8846.

(8) Zhang, H.; Zhang, Y.; Li, Y.; Ahn, S.; Palmore, G. T. R.; Fu, J.; Peterson, A. A.; Sun, S., Cu nanowire-catalyzed electrochemical reduction of CO or CO_{-2}. *Nanoscale* **2019,** *11* (25), 12075-12079.

(9) Liu, H.; Xiang, K.; Liu, Y.; Zhu, F.; Zou, M.; Yan, X.; Chai, L., Polydopamine Functionalized Cu Nanowires for Enhanced CO_2 Electroreduction Towards Methane. *ChemElectroChem* **2018,** *5* (24), 3991-3999.

(10) Wen, J.; Wan, Z.; Hu, X.; Huang, J.; Kang, X., Restructuring of copper catalysts by potential cycling and enhanced two-carbon production for electroreduction of carbon dioxide. *J. CO2 Util.* **2022,** *56*, 101846.

(11) Wang, Y.; Niu, C.; Zhu, Y.; He, D.; Huang, W., Tunable Syngas Formation from Electrochemical CO_2 Reduction on Copper Nanowire Arrays. *ACS Appl. Energy Mater.* **2020,** *3* (10), 9841-9847.

(12) Zhang, Y.; Si, Z.; Du, H.; Deng, Y.; Zhang, Q.; Wang, Z.; Yu, Q.; Xu, H., Selective CO_2 Reduction to Ethylene Over a Wide Potential Window by Copper Nanowires with High Density of Defects. *Inorg. Chem.* **2022,** *61* (50), 20666-20673.

(13) Choi, C.; Kwon, S.; Cheng, T.; Xu, M.; Tieu, P.; Lee, C.; Cai, J.; Lee, H. M.; Pan, X.; Duan, X.; Goddard, W. A.; Huang, Y., Highly active and stable stepped Cu surface for enhanced electrochemical CO_2 reduction to C_2H_4. *Nat. Catal.* **2020,** *3* (10), 804-812.

(14) Han, L.; Tian, B.; Gao, X.; Zhong, Y.; Wang, S.; Song, S.; Wang, Z.; Zhang, Y.; Kuang, Y.; Sun, X., Copper nanowire with enriched high-index facets for highly selective CO_2 reduction. *SmartMat* **2022,** *3* (1), 142-150.

(15) Cui, F.; Yu, Y.; Dou, L.; Sun, J.; Yang, Q.; Schildknecht, C.; Schierle-Arndt, K.; Yang, P., Synthesis of Ultrathin Copper Nanowires Using Tris(trimethylsilyl)silane for High-Performance and Low-Haze Transparent Conductors. *Nano Lett.* **2015,** *15* (11), 7610-7615.

(16) Li, Y.; Cui, F.; Ross, M. B.; Kim, D.; Sun, Y.; Yang, P., Structure-Sensitive CO_2 Electroreduction to Hydrocarbons on Ultrathin 5-fold Twinned Copper Nanowires. *Nano Lett.* **2017,** *17* (2), 1312-1317.

(17) Zhang, Y.; Cai, Z.; Zhao, Y.; Wen, X.; Xu, W.; Zhong, Y.; Bai, L.; Liu, W.; Zhang, Y.; Zhang, Y.; Kuang, Y.; Sun, X., Superaerophilic copper nanowires for efficient and switchable CO_2 electroreduction. *Nanoscale Horiz.* **2019,** *4* (2), 490-494.

(18) Raciti, D.; Mao, M.; Park, J. H.; Wang, C., Mass transfer effects in CO_2 reduction on Cu nanowire electrocatalysts. *Catal. Sci. Technol.* **2018,** *8* (9), 2364-2369.

(19) Cao, L.; Raciti, D.; Li, C.; Livi, K. J. T.; Rottmann, P. F.; Hemker, K. J.; Mueller, T.; Wang, C., Mechanistic Insights for Low-Overpotential Electroreduction of CO_2 to CO on Copper Nanowires. *ACS Catal.* **2017,** *7* (12), 8578-8587.

(20) Raciti, D.; Livi, K. J.; Wang, C., Highly Dense Cu Nanowires for Low-Overpotential CO_2 Reduction. *Nano. Lett.* **2015,** *15* (10), 6829-6835.

(21) Ma, M.; Djanashvili, K.; Smith, W. A., Controllable Hydrocarbon Formation from the Electrochemical Reduction of CO_2 over Cu Nanowire Arrays. *Angew. Chem. Int. Ed.* **2016,** *55* (23), 6680-6684.
